# NRGN inhibits CD8⁺ T cell effector function by downregulating MHC-I expression through the FMR1–IRF8 axis in hepatocellular carcinoma

**DOI:** 10.1186/s13046-026-03783-7

**Published:** 2026-07-15

**Authors:** Dongjie Ye, Jiacheng Qiu, Haoyi Yang, Jinqiao Wu, Xinyu Zhang, Zhu Zhang, Hao Wu, Yuxin Yao, Xiaoqian Wang, Nanhong Tang

**Affiliations:** 1 Department of Hepatobiliary Surgery and Fujian Institute of Hepatobiliary Surgery, Fujian Medical University Union Hospital, Fuzhou, China; 2 Department of Laboratory Medicine, Fujian Medical University Union Hospital, Fuzhou, China; 3 Cancer Center of Fujian Medical University, Fujian Medical University Union Hospital, Fuzhou, China; 4 Key Laboratory of Clinical Laboratory Technology for Precision Medicine (Fujian Medical University), Fujian Province University, Fuzhou, China; 5 Key Laboratory of Gastrointestinal Cancer (Fujian Medical University), Ministry of Education, Fuzhou, China

**Keywords:** Hepatocellular carcinoma, NRGN, CD8⁺ T cells, Antigen presentation, Immune evasion

## Abstract

**Background:**

Neurogranin (NRGN) has been extensively studied as a neuron-enriched synaptic regulatory protein, yet its function in cancer remains largely unexplored. Immune evasion driven by defective antigen presentation and CD8⁺ T cell dysfunction is a hallmark of hepatocellular carcinoma (HCC). This study aimed to determine whether NRGN contributes to immune escape in HCC and to elucidate its underlying molecular mechanism.

**Methods:**

NRGN expression and clinical relevance were analyzed in public datasets and validated in HCC tissues. Transcriptomic and enrichment analyses assessed antigen presentation. Gain- and loss-of-function experiments were performed in human and murine HCC cells, while orthotopic and subcutaneous models with CD8⁺ T cell exhaustion, β2M knockout, and H-2Kb blockade evaluated immune dependence in vivo. RNA stability, RNA pulldown, RIP, co-immunoprecipitation, ubiquitination, and luciferase assays were conducted to define the molecular mechanisms.

**Results:**

NRGN was significantly upregulated in HCC and associated with advanced stage and poor survival, correlating inversely with GZMB⁺ CD8⁺ T cell infiltration. NRGN knockdown enriched MHC-I antigen presentation pathways and restored MHC-I/β2M expression, tumor cell surface antigen presentation, and CD8⁺ T cell cytotoxicity. In vivo, NRGN silencing suppressed tumor growth and prolonged survival in a CD8⁺ T cell-dependent manner, effects reversed by β2m deletion or H-2Kb blockade. Mechanistically, NRGN downregulated IRF8 by reducing its mRNA stability through FMR1 and stabilizing FMR1 protein via inhibition of MYCBP2-mediated ubiquitination, thereby sustaining suppression of antigen presentation.

**Conclusions:**

NRGN suppresses antigen presentation via the FMR1-IRF8-MHC-I axis, thereby impairing CD8⁺ T cell-specific effector functions and promoting immune evasion in HCC. Modulation of the NRGN–IRF8–MHC-I axis could represent a potential strategy for improving immunotherapeutic responses.

**Graphical Abstract:**

**Figure:**
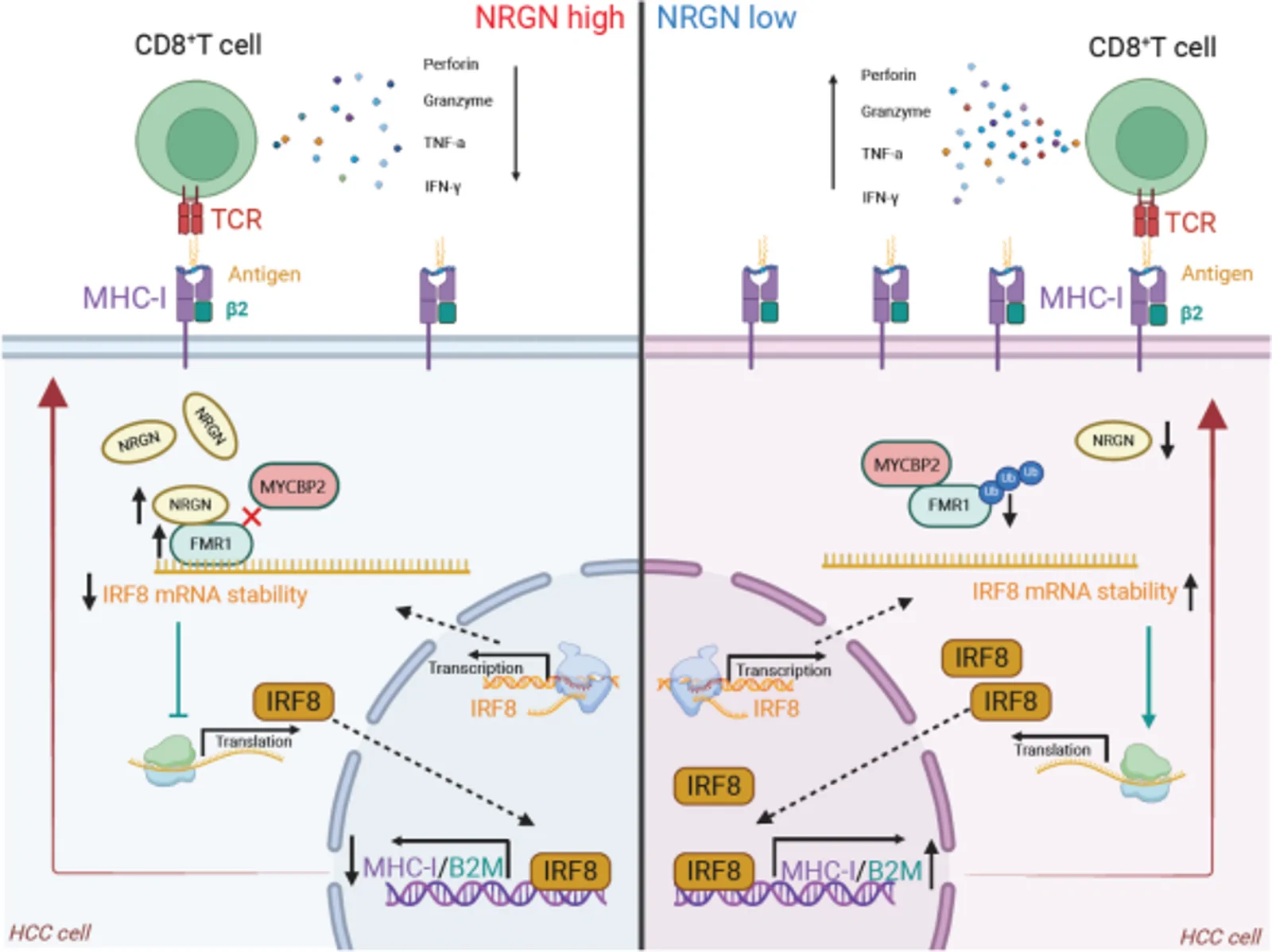

**Supplementary Information:**

The online version contains supplementary material available at https://doi.org/10.1186/s13046-026-03783-7.

## Introduction

Hepatocellular carcinoma (HCC) is one of the most prevalent and lethal malignancies worldwide, characterized by a complex pathogenesis and an overall poor clinical prognosis [1, 2]. Despite advances in therapeutic strategies, including surgical resection, radiofrequency ablation, chemotherapy, and targeted therapies, long-term survival remains unsatisfactory, particularly in patients with advanced-stage disease, who exhibit high rates of tumor recurrence and metastasis [3]. Accumulating evidence indicates that the tumor immune microenvironment (TIME) plays a pivotal role in HCC initiation and progression, and that tumor-mediated suppression of immune surveillance is a major determinant of the limited efficacy of current immunotherapeutic approaches [4, 5].

Defective antigen presentation is considered a central mechanism underlying tumor immune evasion [6, 7]. The expression of major histocompatibility complex class I (MHC-I) molecules on the surface of tumor cells is essential for effective recognition and elimination by CD8⁺ cytotoxic T lymphocytes (CTLs) [8–10]. By presenting intracellularly derived peptide antigens, MHC-I enables the immune system to detect transformed or malignant cells [11, 12]. However, genetic and epigenetic alterations frequently observed in HCC disrupt antigen presentation pathways, resulting in immune escape [13]. In particular, many HCC tumors downregulate MHC-I and its associated components, such as β2-microglobulin (β2M), thereby impairing antigen presentation efficiency and evading CD8⁺ T cell-mediated immune surveillance [14, 15]. This mechanism not only facilitates tumor persistence but also limits the therapeutic efficacy of immune checkpoint blockade. Consequently, elucidating the molecular mechanisms that govern MHC-I expression and antigen presentation is critical for the identification of novel immunotherapeutic targets in HCC.

CD8⁺ T cells serve as the principal effector cells in antitumor immunity, and their activity depends on efficient antigen processing and presentation, antigen-specific recognition, activation, and sustained effector function [4]. In HCC, however, CD8⁺ T cells frequently exhibit functional impairment, manifested by insufficient priming, restricted antigen recognition, and suppressed cytotoxic activity [16]. Tumor cells can attenuate CD8⁺ T cell function through classical immunosuppressive signaling pathways or tumor-intrinsic regulatory mechanisms, thereby promoting immune evasion [17–19]. Despite these observations, the tumor-derived intrinsic factors that regulate antigen presentation and CD8⁺ T cell dysfunction in HCC remain incompletely understood, representing a critical gap in our knowledge of HCC immune biology.

Neurogranin (NRGN) is a calcium-binding protein predominantly expressed in the nervous system, where it plays an essential role in synaptic plasticity and signal transduction. Traditionally regarded as a neuron-specific, synapse-associated molecule, NRGN is primarily involved in the regulation of neuronal signaling and synaptic plasticity, with extensive basic and clinical evidence supporting its roles in learning and memory, as well as in psychiatric and neurodegenerative disorders [20, 21]. Recent studies have reported aberrant NRGN expression in multiple non-neuronal malignancies, implicating its involvement in tumor progression through modulation of oncogenic signaling pathways and the tumor microenvironment (TME) [22–24]. Our previous work demonstrated that NRGN suppresses CD8⁺ T cell effector function by regulating immunosuppressive factors within the TME, including reduced cytokine production and increased expression of exhaustion markers [23]. These findings suggest that NRGN may function as an immunomodulatory factor in HCC, potentially influencing tumor antigen presentation and CD8⁺ T cell-mediated antitumor immunity. Consistently, preliminary bioinformatic analyses and experimental observations indicate that NRGN is highly expressed in HCC tissues and correlates with CD8⁺ T cell infiltration and patient prognosis.

In this study, we sought to delineate the role of NRGN in HCC immune evasion from a tumor cell-intrinsic perspective. We systematically investigated the impact of NRGN on tumor antigen presentation and CD8⁺ T cell antitumor function. Our findings demonstrate that NRGN markedly suppresses the expression of MHC-I and β2M in tumor cells, leading to impaired CD8⁺ T cell activation and cytotoxicity, thereby facilitating immune escape. Collectively, this work provides new insights into the mechanisms of immune evasion in HCC and establishes a conceptual framework for the development of future immunotherapeutic strategies targeting tumor-intrinsic regulation of antigen presentation.

## Methods

### Clinical specimens and HCC tissue microarray

Tumor tissues from 12 HCC patients were collected at the Fujian Medical University Union Hospital, with all tumor types confirmed by pathological examination. Resected tissues were fixed, embedded in paraffin, sectioned, and stored at room temperature. None of the patients received radiotherapy or chemotherapy prior to surgery, and informed consent was obtained from all participants. Sample collection and experimental procedures were approved by the Institutional Medical Ethics Committee of our hospital (Approval No. 2021KJCX008). Additionally, a tissue microarray (TMA) containing 80 paired HCC and adjacent non-tumor tissues (LvcSur2201) was purchased from Hunan Aifang Biotechnology Co., Ltd., with informed consent obtained from all patients. All studies were conducted in accordance with the Declaration of Helsinki and the Istanbul Declaration.

### Experimental animals

Specific-pathogen-free (SPF) male C57BL/6 mice, aged 6–8 weeks, were obtained from Shanghai SLAC Laboratory Animal Co., Ltd. Mice were housed in the Animal Center of Fujian Medical University under SPF conditions, with cages, feed, bedding, and drinking water sterilized by autoclaving. Bedding was replaced twice weekly. All animal experiments were approved by the Animal Ethics Committee of Fujian Medical University (Approval No. IACUC FJMU 2024 − 0377) and were conducted in accordance with the approved protocols. Animals were maintained and handled following the guidelines for the care and use of laboratory animals published by the U.S. National Institutes of Health (NIH).

### Plasmids and bacterial strains

Human and mouse NRGN shRNA plasmids (Ng_KD), human and mouse NRGN overexpression plasmids (Ng_OE), human IRF8 overexpression plasmid (IRF8_OE), human FMR1 overexpression plasmid (FMR1_OE), human IRF8 knockdown plasmid (IRF8_KD), human FMR1 knockdown plasmid (FMR1_KD), human MYCBP2 knockdown plasmid (MYCBP2_KD), human and mouse IRF8 CRISPR-Cas9 knockout plasmids (IRF8_KO), and mouse β2m CRISPR-Cas9 knockout plasmid (β2m_KO) were obtained from Tsingke Biotechnology Co., Ltd. Luciferase reporter plasmids including HLA-A-Luc, B2M-Luc, IRF8-Luc, and the sea kidney luciferase control plasmid pRL-TK were also purchased from Tsingke Biotechnology Co., Ltd. Chemically competent *Escherichia coli* DH5α cells were purchased from Beijing Zhuangmeng Biotechnology Co., Ltd.

### Cell culture

HEK-293T cells and human HCC cell lines Huh7, Hep3B, Hep1-6 and murine cytotoxic T-cell line CTLL-2 were obtained from the American Type Culture Collection (ATCC), and the mouse HCC cell line Hep-53.4 was obtained from Cytion GmbH, Germany. HEK-293T, Huh7, Hep3B, Hep1-6 and Hep-53.4 were maintained in Dulbecco’s Modified Eagle Medium (DMEM; 10566016, Thermo Fisher, MA) supplemented with 12% (v/v) fetal bovine serum (FBS; 16140071, Thermo Fisher) and 100 U/mL penicillin-streptomycin (15140148, Thermo Fisher) at 37 °C in a humidified incubator with 5% CO₂. CTLL-2 cells were maintained in RPMI 1640 medium supplemented with 10% FBS, penicillin–streptomycin mixed solution, and recombinant human IL-2 (100 U/mL). HEK-293T cells were used for lentiviral packaging. All cell lines were routinely tested for mycoplasma contamination using the MycoSEQ Mycoplasma Detection Kit (4460623, Thermo Fisher) to ensure culture integrity.

### RNA-seq

RNA sequencing was performed by Tsingke Biotechnology Co., Ltd, including RNA extraction, quality control, library preparation, and sequencing. Raw sequencing reads were subjected to quality control and aligned to the reference genome, followed by gene expression quantification. Differential gene expression analysis was performed using standard statistical methods with predefined thresholds (e.g., |log2 fold change| > 1 and p-value < 0.05). Functional enrichment analyses, including Gene Ontology (GO) and Kyoto Encyclopedia of Genes and Genomes (KEGG) pathway analyses, were subsequently conducted to interpret the biological significance of differentially expressed genes.

### Functional enrichment and interaction analysis

Differential gene expression analysis was performed on transcriptomic data using R packages. Subsequently, GO enrichment analysis of differentially expressed genes was conducted using the clusterProfiler R package, and gene annotations were obtained from org.Hs.eg.db [25]. A false discovery rate (FDR)-adjusted *P* value of < 0.05 was considered statistically significant. In addition, Gene Set Enrichment Analysis (GSEA) was performed to assess hallmark pathways using the h.all.v7.4.symbols.gmt gene set downloaded from the Molecular Signatures Database (MSigDB) [26].

### siRNA transfection

siRNA targeting human *NRGN* and mouse *Nrgn* were obtained from Tsingke Biotechnology Co., Ltd. According to the instructions, we introduced an appropriate amount of siRNA into cells through transfection reagents. After 36 h, we used Western blotting to detect the protein expression.

### RNA extraction and quantitative real-time PCR

Total RNA was extracted and purified from cells or tissues using the FastPure Cell/Tissue Total RNA Isolation Kit (RC101-01, Vazyme, Nanjing, China), followed by reverse transcription using HiScript II Q RT SuperMix for qPCR (R223-01, Vazyme). qPCR was performed on an Applied Biosystems 7500 and 7500 Fast Real-Time PCR System using ChamQ SYBR qPCR Master Mix (Q311-02, Vazyme). The PCR cycling conditions were as follows: initial denaturation at 95 °C for 30 s, followed by 40 cycles of 95 °C for 10 s, 63 °C for 10 s, and 72 °C for 30 s. Melting curves were generated according to the manufacturer’s standard protocol. Relative gene expression levels were calculated using the 2^(-ΔΔCT) method with GAPDH as the internal control. Primer sequences are listed in Table S1.

### Total protein extraction from clinical HCC tissues

Fresh HCC tissues obtained from surgical resection were immediately snap-frozen in liquid nitrogen and stored at -80 °C. Prior to protein extraction, tissues were briefly thawed on ice, and blood and necrotic regions were removed. Approximately 30–50 mg of tissue was placed into pre-chilled microcentrifuge tubes. Cold RIPA lysis buffer supplemented with protease and phosphatase inhibitors (volume 8–10× tissue weight) was added under low-temperature conditions. Tissues were homogenized on ice using a tissue homogenizer and lysed on ice for 30 min with intermittent gentle mixing. Lysates were then centrifuged at 12,000–14,000 × g for 15 min at 4 °C, and the supernatants were collected as total protein extracts. Protein concentrations were determined using a BCA Protein Assay Kit (WB6501, NCM Biotech) and normalized. For subsequent analyses, 5× SDS-PAGE loading buffer (WB2001, NCM Biotech, Suzhou, China) was added, and samples were denatured at 95 °C for 5 min. Samples were stored at -20 °C for short-term use or -80 °C for long-term storage.

### Western blotting

Cells were lysed in RIPA buffer (WB3100, NCM Biotech) and centrifuged at 12,000 rpm for 30 min at 4 °C. Protein concentrations in the supernatants were determined using a BCA Protein Assay Kit (WB6501, NCM Biotech). Equal amounts of protein were loaded onto 12% polyacrylamide gels for electrophoretic separation and subsequently transferred onto polyvinylidene difluoride (PVDF) membranes (IPVH00010, Millipore, MA). Membranes were blocked with QuickBlock™ blocking buffer (P0252, Beyotime, Shanghai, China) for 15 min. Following blocking, membranes were incubated with primary antibodies overnight at 4 °C and then with HRP-conjugated goat anti-rabbit IgG (H + L) secondary antibody (SA00001-2, Proteintech, Wuhan, China) for 1 h at room temperature. Protein signals were visualized using an enhanced chemiluminescence (ECL) kit (P10300, NCM) and imaged on a ChemiDoc MP Imaging System. The primary antibodies used included Neurogranin (10440-1-AP, Proteintech), MHC-I (A8754, Abclonal, Wuhan, China), Β2M (13511-1-AP, Proteintech), Ki67 (27309-1-AP, Proteintech), CD8 (ab217344, Abcam), GZMB (ab 255598, Abcam), IRF8 (18977-1-AP, Proteintech), MYCBP2 (HA722030, HUABIO, Hangzhou, China), FMR1 (66548-1-Ig, Proteintech), Rabbit IgG (30000-0-AP, Proteintech), Mouse IgG (B900620, Proteintech), HA (51064-2-AP, Proteintech), His (10001-0-AP, Proteintech), DYKDDDDK(Flag) (80801-2-RR, Proteintech) and GAPDH (GB15004-100, Servicebio, Wuhan, China).

### Immunoprecipitation

Protein lysates were prepared as described for Western blotting. For each sample, 5% of the total lysate was reserved as input. The remaining lysates were incubated overnight at 4 °C with antibodies against NRGN, FMR1, His, HA, or Flag, followed by incubation with Pierce Protein A/G magnetic beads (P2055, Beyotime) for 1 h at room temperature with rotation. Bead-bound protein complexes were separated by electrophoresis on 12% polyacrylamide gels and transferred onto PVDF membranes. Subsequent steps were performed as described for Western blotting.

### Orthotopic HCC mouse model

Male C57BL/6J mice (6 weeks old, 18–20 g) were maintained under SPF conditions with a 12 h light/12 h dark cycle. For the orthotopic liver tumor model, 2 × 10⁶ Hep-53.4 mouse HCC cells stably transfected with Ng_KD or control vector were suspended in 25 µL of Matrigel (G4131, Servicebio) and implanted into the left liver lobe of anesthetized C57BL/6J mice via surgical exposure. For the H-2Kb blocking experiments, three days after orthotopic transplantation, mice were randomly divided into four groups: Ng_Ctrl + IgG, Ng_Ctrl + αH-2Kb, Ng_KD + IgG, and Ng_KD + αH-2Kb. Mice received intraperitoneal injections of 200 µg anti-H-2Kb antibody (BE0172, BioXcell, New Hampshire) or IgG isotype control (BE0086, BioXcell) twice per week. For the PD-1 treatment experiments, seven days after orthotopic transplantation, mice were randomly assigned to four groups: Ng_Ctrl + IgG, Ng_Ctrl + αPD-1, Ng_KD + IgG, and Ng_KD + αPD-1. Mice received intraperitoneal injections of 200 µL anti-PD-1 antibody (A2122, Selleck, Houston) or IgG isotype control (A2123, Selleck) twice per week. At the end of the experiments, mice were sacrificed and tumor tissues were collected for downstream analyses.

### Subcutaneous HCC mouse model

Male C57BL/6J mice (6 weeks old, 18–20 g) were maintained under SPF conditions with a 12 h light/12 h dark cycle. For the subcutaneous tumor model, 1 × 10⁶ Hep-53.4 mouse HCC cells stably transfected with Ng_KD or control vector were injected subcutaneously into the left flank of each mouse. Tumor dimensions were measured every 5 days using calipers, and tumor volumes were calculated using the formula: $\:\mathrm{Volume}=(\mathrm{length}\times\:{\mathrm{width}}^{2})/2$. At the end of the experiments, mice were sacrificed, and tumors were excised, weighed, and photographed. For H-2Kb blocking experiments, three days after subcutaneous inoculation, mice were randomly divided into four groups: Ng_Ctrl + IgG, Ng_Ctrl + αH-2Kb, Ng_KD + IgG, and Ng_KD + αH-2Kb. Mice received intraperitoneal injections of 200 µg anti-H-2Kb antibody (BE0172, BioXcell, New Hampshire) or IgG isotype control (BE0086, BioXcell) twice per week. For PD-1 treatment experiments, seven days after tumor inoculation, mice were randomly assigned to four groups: Ng_Ctrl + IgG, Ng_Ctrl + αPD-1, Ng_KD + IgG, and Ng_KD + αPD-1. Mice received intraperitoneal injections of 200 µL anti-PD-1 antibody (A2122, Selleck, Houston) or IgG isotype control (A2123, Selleck) twice per week. At the conclusion of the experiments, mice were sacrificed, and tumor tissues were collected for downstream analyses.

### Collection and processing of mouse liver specimens

Mice were euthanized with carbon dioxide, and the left ventricle of the heart was perfused with PBS using a 25G butterfly needle and a 50 mL syringe. Excised livers were cut into 1–3 mm pieces and digested at 37 °C for 30 min in RPMI 1640 medium containing 0.4 mg/mL collagenase type IV (17104019, Thermo Fisher, MA), 10 mg/mL DNase I (18047019, Thermo Fisher), and 10% fetal bovine serum (FBS), with continuous shaking. The enzymatic reaction was terminated by adding 5 mM EDTA (005500, Thermo Fisher). To homogenize the tissue, samples were repeatedly aspirated through a 20G needle attached to a 5 mL syringe until a uniform suspension was obtained. The homogenate was filtered through a 70 μm cell strainer and centrifuged at 400 × g for 8 min at 4 °C to pellet cells and myelin debris. Myelin was removed using a density gradient centrifugation: the pellet was resuspended in PBS containing 30% Percoll (BS909, Biosharp, Beijing, China) and centrifuged at 1,592 × g for 30 min at 4 °C in a 50 mL fixed-angle rotor, maintaining consistent homogenization speed. The upper white layer (myelin) was discarded, and the intermediate transparent layer was collected, filtered through a 70 μm strainer, washed with PBS, and centrifuged at 400 × g for 8 min at 4 °C. The resulting single-cell suspension was used for flow cytometry analysis.

### Multiparametric immunofluorescence (mIF) staining

mIF was conducted using a four-color multiple fluorescence immunohistochemical staining kit (RS0035, Immunoway, TX) that employs the tyramide signal amplification technique. The staining process was carried out in strict accordance with the manufacturer’s instructions. Subsequently, the stained images were meticulously captured using a fluorescence microscope.

### Flow cytometry analysis

To assess the expression of cell surface markers, cells were incubated with the indicated fluorophore-conjugated antibodies in Flow Cytometry Staining Buffer (00-4222-26, Thermo Fisher) at 4 °C for 30 min. Following incubation, cells were washed twice with PBS and resuspended in staining buffer. Data acquisition was performed on a FACSCelesta™ flow cytometer, and analyses were conducted using FlowJo software.

The antibodies used included: Alexa Fluor^®^ 700 anti-mouse CD45 (Biolegend, #103128, CA), Brilliant Violet 510 M anti-human CD3 (Biolegend, #317331), Brilliant Violet 650™ anti-mouse CD4 (Biolegend, #100555), APC-Cy™7 anti-mouse CD8a (BD Biosciences, #557654, CA), BD Horizon™ Fixable Viability Stain 575 V (BD Biosciences, #565694), PE anti-human/mouse Granzyme B recombinant antibody (Biolegend, #372207), FITC anti-mouse IFN-γ (Biolegend, #505805), and BD Horizon™ BB700 anti-mouse TNF (BD Biosciences, #566510), Alexa Fluor^®^ 488 anti-human HLA-A, B,C(Biolegend, #311413), APC anti-human β2-microglobulin (Biolegend, #316311), FITC anti-mouse H-2Kb/H-2Db (Biolegend, #114605), APC anti-mouse β2-microglobulin (Biolegend, #154505), PE anti-mouse H-2Kb bound to SIINFEKL Antibody (Biolegend, #141604), Brilliant Violet 785™ anti-mouse CD69 Antibody (Biolegend, #104543), FITC anti-mouse NK-1.1 (Biolegend, #108705).

### Immunohistochemistry (IHC)

Tumor tissues were fixed in 4% paraformaldehyde, dehydrated, and embedded in paraffin before being sectioned at 5 µm thickness. Following antigen retrieval, sections were blocked with 10% bovine serum albumin and incubated overnight at 4°C with primary antibodies at their optimized dilutions. Subsequently, sections were incubated with HRP-conjugated secondary antibodies for 1 hour at room temperature. Signal development was performed using 3,3’-diaminobenzidine (DAB, G1212, Servicebio), and nuclei were counterstained with hematoxylin. All stained sections were examined and imaged using a light microscope (Carl Zeiss, Germany).

### Hematoxylin and Eosin (H&E) staining

For H&E staining, paraffin-embedded tumor sections from mice were deparaffinized in xylene and rehydrated through a graded ethanol series. Sections were stained with hematoxylin (AR1180-1, Boster, Wuhan, China) for 4 min at room temperature to visualize nuclei, followed by eosin (AR1180-2, Boster) for 1 min to stain cytoplasm. Stained sections were rinsed under running water for 10 min and subsequently imaged using a light microscope. Morphological analyses were then performed on the acquired images.

### Dual-Luciferase Reporter assay (DLR)

Human-derived reporter plasmids, including pGL3-HLA-A promoter-Luc, pGL3-B2M promoter-Luc, and pGL3-IRF8 promoter-Luc, were obtained from Tsingke Biotechnology Co., Ltd. These reporter plasmids were co-transfected with NRGN or IRF8 expression plasmids and the pGL3 basic vector into cells using Lipofectamine 3000 (L3000075, Thermo Fisher) according to the manufacturer’s instructions. The pRL-TK plasmid (Promega) was included as an internal control. After 36 h of transfection, cells were lysed, and luciferase activities were measured using the Dual-Luciferase Reporter Assay System (E1910, Promega, WI) following the manufacturer’s protocol.

### Peripheral Blood Mononuclear Cell (PBMC) isolation

Peripheral whole blood (7.5 mL) was collected and diluted with an equal volume of PBS, followed by thorough mixing. Fifteen milliliters of Ficoll density gradient medium were preloaded into a 50 mL centrifuge tube, and the diluted blood was carefully layered on top of the Ficoll. Samples were centrifuged at 2,000 rpm for 20 min at 25 °C with both acceleration and deceleration set to 0. Following centrifugation, the distinct mononuclear cell layer at the plasma-Ficoll interface was carefully collected and transferred to a new tube. Cells were washed twice with PBS and finally resuspended in T-cell culture medium at a density of 2 × 10⁶ cells per 2 mL dish. Recombinant IL-2 was added to the culture medium at a final concentration of 10 ng/mL to maintain T-cell survival and proliferation.

### Genotyping of OT-I mice by agarose gel electrophoresis

Genomic DNA amplified by polymerase chain reaction (PCR) from mouse tail samples was used for OT-I mouse genotyping following the instructions of a commercial rapid genotyping kit (D7283S, Beyotime). A 1% (w/v) agarose gel was prepared in 1×TAE buffer, heated to dissolve, cooled to approximately 60 °C, and supplemented with ethidium bromide before being cast into a gel tray with combs. Once solidified, the gel was placed in an electrophoresis chamber containing 1×TAE buffer. PCR products were mixed with 6× DNA loading dye and loaded into the wells. Electrophoresis was performed at a constant voltage of 140 V until the bromophenol blue front migrated to approximately 1 cm from the gel bottom. DNA bands were visualized under ultraviolet light using a gel documentation system. OT-I mouse genotypes were determined by comparing band patterns with a DNA ladder.

### Co-culture assays

For co-culture experiments with Hep-53.4_OVA tumor cells and OT-I mouse CD8⁺ T cells, OT-I transgenic mice were kindly provided by Dr. Banglun Pan (Mengchao Hepatobiliary Hospital of Fujian Medical University). 36 h prior to co-culture, tumor cells were seeded into 24-well plates (2 × 10⁴ cells/well) and 96-well plates (3 × 10³ cells/well). CD8⁺ T cells were isolated from the spleens of OT-I mice by flow cytometric sorting and were activated in RPMI 1640 medium containing 10% fetal bovine serum with αCD3 (5 µg/mL) and αCD28 (2 µg/mL) antibodies at 37 °C with 5% CO₂ for 36 h. Activated T cells were then added to the tumor cells in the 24- and 96-well plates at various effector-to-target ratios for co-culture.

After 24 h of co-culture, suspended CD8⁺ T cells were removed, and adherent tumor cells were washed 3 times with PBS. Tumor cell apoptosis was assessed by flow cytometry using an apoptosis detection kit according to the manufacturer’s instructions (E-CK-A217, Elabscience, TX). Tumor cell viability was measured using the Cell Counting Kit-8 assay (CCK8, BMU106, Abbkine, Wuhan, China). CD8⁺ T cell-mediated cytotoxicity was evaluated using a lactate dehydrogenase (LDH) release assay (G1610-100T, Servicebio).

To assess H-2Kb-restricted cytotoxicity, Hep-53.4_OVA cells with different NRGN expression levels were incubated with either anti-H-2Kb-SIINFEKL antibody or IgG isotype control (100 µg/mL) at 4 °C for 30 min. Activated OT-I CD8⁺ T cells were then co-cultured with tumor cells at a 5:1 effector-to-target ratio for 12 h. Cytotoxic activity of CD8⁺ T cells was subsequently analyzed by flow cytometry.

### Apoptosis assay

Tumor cell apoptosis was assessed using Annexin V-FITC/7-AAD (E-CK-A217, Elabscience) staining according to the manufacturer’s instructions. Cells were harvested by trypsinization, washed once with PBS, and collected by centrifugation at 300 ×g for 5 min. A total of 1–5 × 10⁵ cells were resuspended in 100 µL of 1× Annexin V Binding Buffer, followed by incubation with Annexin V-FITC and 7-AAD at room temperature in the dark for 15–20 min. Subsequently, 400 µL of 1× Binding Buffer was added, and samples were analyzed immediately by flow cytometry. If immediate analysis was not possible, samples were kept on ice in the dark and analyzed within 1 h.

### CD8⁺ T cell migration

For the transwell migration assay, activated CD8^+^ T cells (CTLL-2) were washed and resuspended in serum-free RPMI-1640 medium. A total of 2 × 105 CD8^+^ T cells in 200 µL medium were seeded into the upper chamber of a 24-well transwell insert (6.5 mm diameter with 5.0 μm polycarbonate membrane; #14331, LABSELECT, Zhejiang, China). The lower chamber contained 600 µL of cell culture supernatant collected from control or experimental conditions as a chemoattractant. After incubation for 24 h at 37 °C in a humidified atmosphere with 5% CO2, the cells that had migrated into the lower chamber were collected, stained with anti-CD8 antibody (BD Biosciences, #557654) and counting beads (Biolegend, # 424902), and quantified by flow cytometry [27].

### RNA pull-down assay

The RNA pulldown assay was conducted using an RNA pulldown kit (Bes5102, BersinBio, Guangzhou, China) according to the manufacturer’s protocol. In brief, 2 × 10⁷ cells were lysed and incubated overnight at 4 °C with 3 µg of biotin-labeled oligonucleotide probes targeting the exogenous IRF8 3′ UTR, 5′ UTR + CDS, and coding sequence (BersinBio). Streptavidin-coated magnetic beads were subsequently added, and the lysates were incubated for an additional 2 h at room temperature. After thorough washing, pulled-down proteins were subjected to Western blot analysis to detect RNA-associated proteins.

### RNA immunoprecipitation (RIP) assay

The RNA immunoprecipitation assay was conducted using an RIP kit (Bes5101, BersinBio) according to the manufacturer’s protocol. RIP assays were performed to examine RNA-protein interactions using a polysome-based protocol. Briefly, approximately 2 × 10⁷ cells were lysed in polysome lysis buffer supplemented with protease inhibitors and RNase inhibitor under RNase-free conditions. Lysates were treated with DNase at 37 °C to remove genomic DNA, followed by reaction termination and clarification by centrifugation. Protein A/G magnetic beads were equilibrated with lysis buffer. Cell lysates were divided into input, IP, and IgG control groups. The IP group was incubated with FMR1 (66548-1-Ig, Proteintech) antibody against the target protein, while the IgG group received an equal amount of control IgG (B900620, Proteintech), followed by overnight incubation at 4 °C with gentle rotation. Subsequently, equilibrated protein A/G beads were added and incubated for an additional 1 h to capture RNA-protein complexes. Beads were washed sequentially with polysome washing buffers to reduce nonspecific binding, and RNA-protein complexes were eluted using polysome elution buffer. RNA was extracted from input, IP, and IgG samples using TRIzol reagent and precipitated with ethanol. Purified RNA was dissolved in RNase-free water and stored at -80 °C. Enrichment of IRF8 mRNA was analyzed by RT-PCR or qRT-PCR.

### mRNA stability assay (actinomycin D treatment)

To assess the effect of NRGN on IRF8 mRNA stability, HCC cells with or without NRGN manipulation were treated with actinomycin D (ActD, GC16866, GLPbio, CA) at a final concentration of 5 µg/mL to inhibit transcription. Cells were harvested at 0, 1, 3, 6, and 9 h after ActD treatment, and total RNA was extracted for qRT-PCR analysis. IRF8 mRNA levels at each time point were normalized to those at 0 h, and the decay kinetics were compared between control and NRGN-manipulated cells to evaluate changes in mRNA stability.

### Protein degradation assays

For cycloheximide (CHX) chase experiments, cells were treated with 10 µg/mL CHX (HY-12320, MedChemExpress, NJ) for 0, 1, 3, 6, 9, and 12 h. To investigate protein degradation pathways, cells were treated with the proteasome inhibitor MG132 (10 µM, T2154, TargetMol, MA) for 8 h. Following treatment, cells were collected and lysed on ice, and protein levels were analyzed by western blotting. For ubiquitination (Ub) assays, plasmids expressing NRGN and HA-Ub were transfected into HCC cells. Cells were cultured in the presence of MG132 to enhance ubiquitination of FMR1. Ubiquitinated FMR1 was subsequently immunoprecipitated using an anti-FMR1 antibody and analyzed as described above.

### Statistical analysis

All data were analyzed using GraphPad Prism v.10 or R software and are presented as mean ± standard deviation (χ ± s). For comparisons between two groups, an unpaired Student’s t-test was used when data were normally distributed with equal variance, whereas the Wilcoxon rank-sum test was applied when data were normally distributed but variances were unequal. Correlations between two variables were assessed using Pearson correlation analysis. Overall survival was analyzed using the log-rank test. Flow cytometry data were analyzed with FlowJo v.10. Statistical significance was defined as *P* < 0.05.

## Result

### High expression of NRGN in HCC and its regulation of antigen presentation and T cell infiltration

**Fig. 1:**
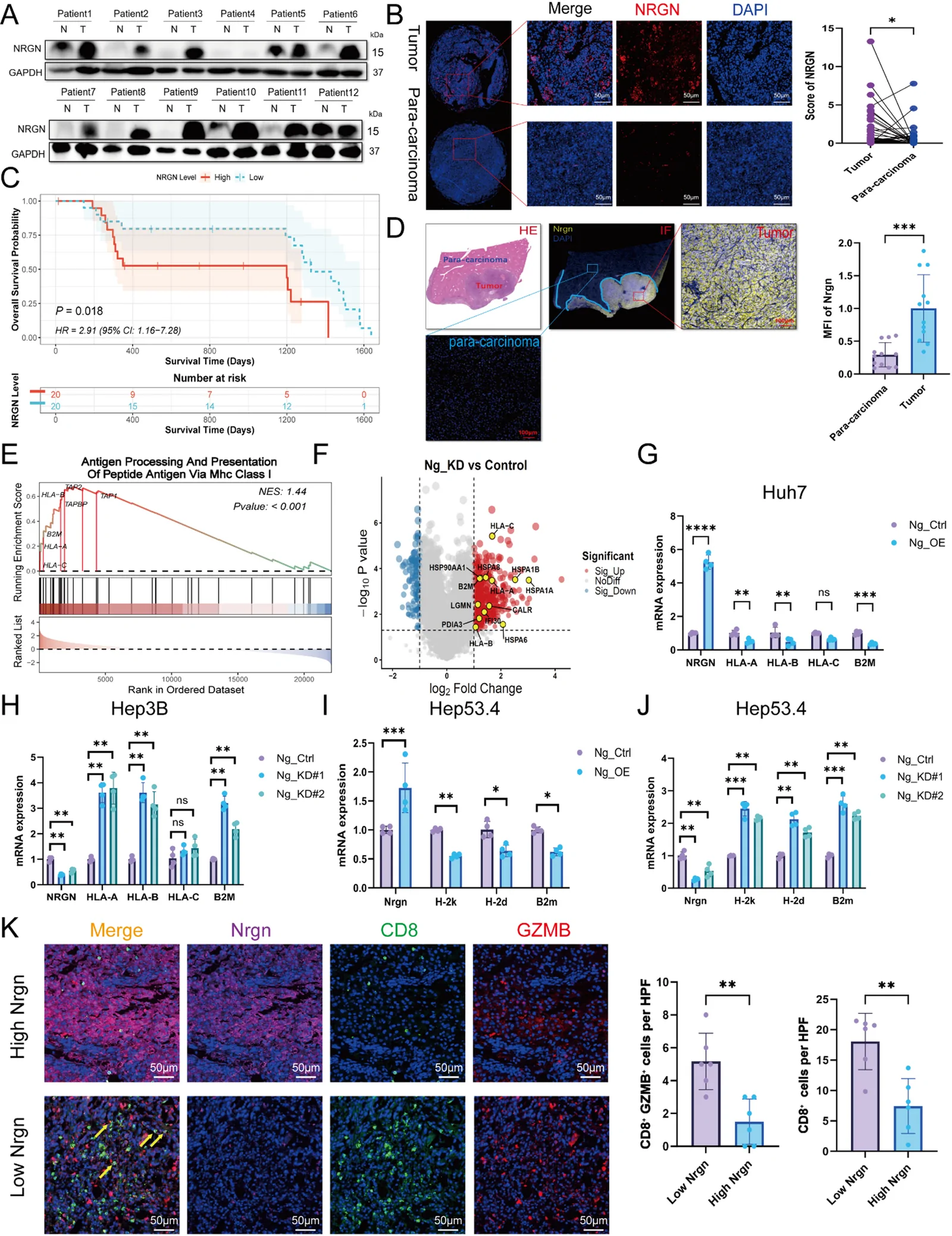
NRGN is highly expressed in HCC and correlates with immunosuppression and poor prognosis. **A** Western blot analysis of NRGN expression in tumor tissues and matched adjacent normal tissues (*n* = 12). **B** Immunofluorescence analysis of NRGN expression in tumor and adjacent non-tumor tissues from a HCC TMA. **C** Survival analysis of HCC patients from the TMA stratified by NRGN expression levels (upper quartile vs. lower quartile). **D** Immunofluorescence validation of NRGN expression in tumor and matched adjacent normal tissues from a mouse orthotopic HCC model (*n* = 12). **E** GSEA based on differentially expressed genes from transcriptomic profiling. **F** Volcano plot of differentially expressed genes from transcriptomic analysis. **G**, **H** qPCR analysis of key antigen-presentation-related genes following NRGN manipulation in Huh7 (**G**) and Hep3B (**H**) cells (*n* = 3). **I**, **J** qPCR analysis of key antigen-presentation-related genes in Hep53.4 mouse HCC cells following NRGN overexpression (**I**) or knockdown (**J**) (*n* = 3). **K** Immunofluorescence staining of tumor tissues from the mouse orthotopic HCC model, quantifying CD8⁺ T cells and GZMB⁺CD8⁺ T cells in NRGN high- vs. low-expression groups. *P < 0.05; **P < 0.01; ***P < 0.001; ****P < 0.0001; ns, not significant

Our previous bioinformatic analyses, supported by experimental validation, revealed that NRGN is differentially expressed across multiple tumor types and exhibits a progressive upregulation in HCC with increasing tumor grade and stage (Fig. S1A, S1B). Moreover, NRGN expression in HCC tissues was significantly higher than in paired adjacent non-tumor tissues (Fig. S1C). To further characterize NRGN expression in HCC, we performed Western blot analysis on collected clinical HCC and paired adjacent tissues. NRGN levels were markedly elevated in tumor tissues compared with non-tumor counterparts (Fig. 1A). Multiplex immunofluorescence staining and TMA analyses of clinical HCC samples further confirmed that NRGN expression was significantly higher in tumor tissues than in adjacent tissues (Fig. 1B). Survival analysis demonstrated that patients with high NRGN expression had significantly shorter overall survival (Fig. 1C). Consistently, analysis of The Cancer Genome Atlas (TCGA) HCC cohort also revealed that elevated NRGN expression was significantly associated with poor prognosis (Fig. S1D).

In vivo, analysis of a murine orthotopic liver tumor model revealed that NRGN expression was markedly higher in tumor tissues compared with adjacent non-tumor tissues, as determined by immunofluorescence staining (Fig. 1D). To explore the potential impact of NRGN on the immune microenvironment in HCC, NRGN was knocked down in Hep3B cells, followed by transcriptomic profiling (Fig. S1E-S1G). GSEA of differentially expressed genes between control and NRGN knockdown groups showed significant enrichment of pathways associated with MHC-I-mediated antigen presentation in the NRGN knockdown group (Fig. 1E). Volcano plot analysis further indicated that most antigen presentation-related genes were upregulated upon NRGN knockdown (Fig. 1F, Fig. S1H). GO enrichment analysis consistently demonstrated that biological processes related to antigen presentation were significantly enriched in the NRGN knockdown group (Fig. S1I).

To validate the transcriptomic results, NRGN was either overexpressed or silenced in Huh7 (Fig. 1G) and Hep3B cells (Fig. 1H), and qPCR was used to assess mRNA levels of antigen presentation-related genes, including HLA-A, HLA-B, HLA-C, and B2M. Compared with controls, NRGN overexpression significantly reduced mRNA expression of these genes, whereas NRGN knockdown led to their upregulation. Similarly, in the mouse HCC cell line Hep53.4, modulation of Nrgn affected the mRNA expression of H-2 K, H-2D, and B2m in a manner consistent with human HCC cell lines (Fig. 1I and J). At the in vivo level, analysis of orthotopic liver tumors revealed a significant negative correlation between Nrgn expression and infiltration of GZMB⁺CD8⁺ T cells (Fig. 1K), where GZMB serves as a key cytotoxic effector molecule released by activated CD8⁺ T cells, mediating target cell apoptosis via the perforin/granzyme pathway and thus representing a marker of their activation and effector function [28]. This indicates that NRGN may contribute to immune evasion in HCC by suppressing antigen presentation and cytotoxic T cell infiltration. We further performed CD8⁺ T cell migration assays and found that the observed differences in infiltration were not attributable to altered chemotaxis (Fig. S1J).

### Tumor-intrinsic NRGN drives HCC progression by impairing antigen presentation and consequently reducing CD8⁺ T cell activation

**Fig. 2:**
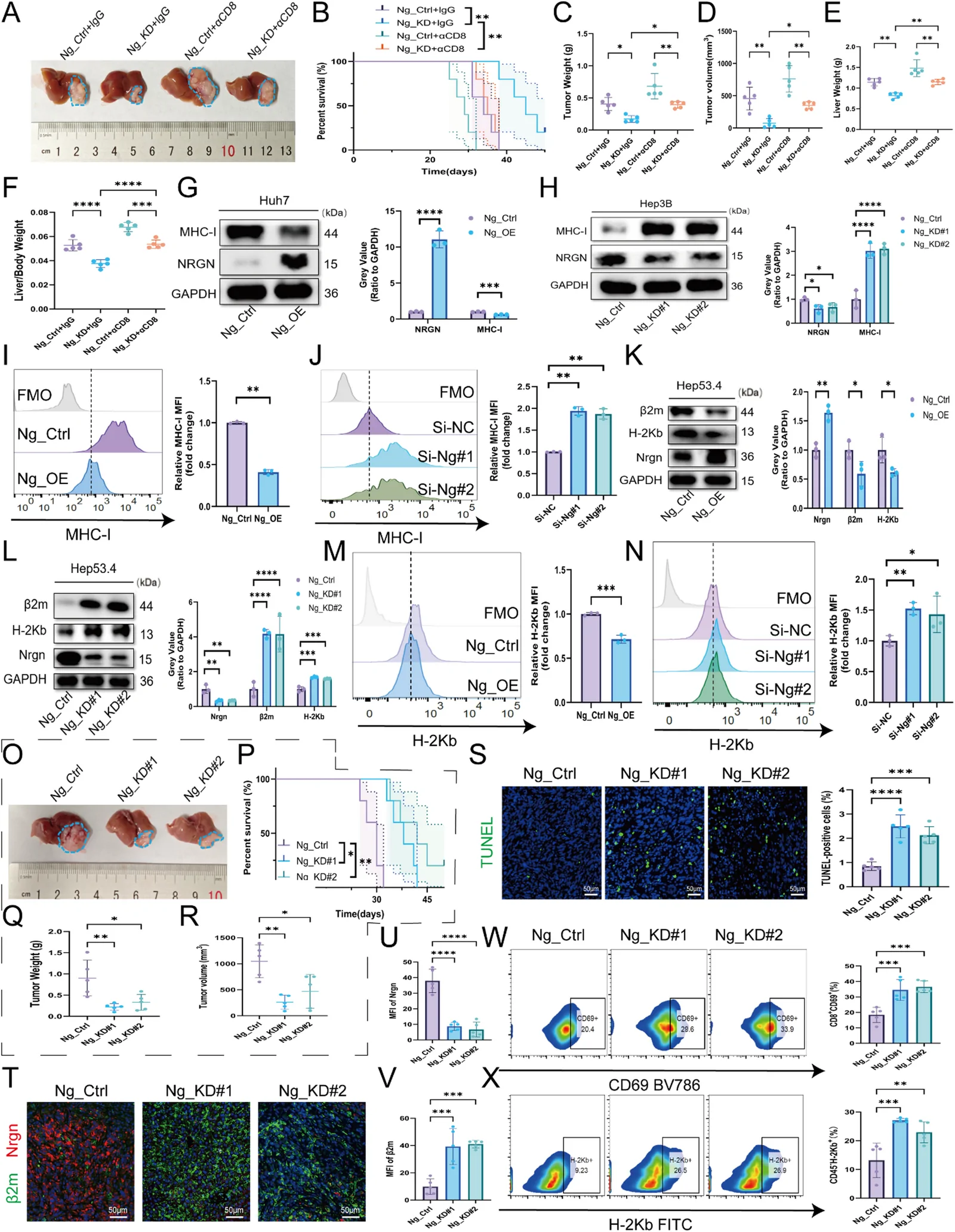
Tumor-intrinsic NRGN suppresses MHC-I antigen presentation to evade CD8⁺ T cell immune surveillance in HCC. **A** Representative images of orthotopic liver tumors in C57BL/6 mice following NRGN manipulation, with or without CD8⁺ T cell exhaustion (*n* = 5). **B**-**F** Quantification of mouse survival (**B**), tumor weight (**C**), tumor volume (**D**), liver weight (**E**), and liver weight/body weight ratio (**F**) in the orthotopic HCC model. **G**, **H** Western blot analysis of MHC-I expression in Huh7 (**G**) and Hep3B (**H**) cells after NRGN manipulation (*n* = 3). **I**, **J** Flow cytometry analysis of cell surface MHC-I levels in Huh7 (**I**) and Hep3B (**J**) cells following NRGN intervention (*n* = 3). **K**, **L** Western blot analysis of H-2Kb and β2m expression in Hep-53.4 cells following NRGN overexpression (**K**) or knockdown (**L**) (*n* = 3). **M**, **N** Flow cytometry analysis of cell surface H-2Kb levels in Hep-53.4 cells following NRGN overexpression (**M**) or knockdown (**N**) (*n* = 3). **O** Representative images of orthotopic liver tumors in C57BL/6 mice generated with Nrgn-knockdown Hep-53.4 cells (*n* = 5). **P**-**R** Quantification of mouse survival (**P**), tumor weight (**Q**), and tumor volume (**R**) in the Nrgn-knockdown orthotopic HCC model. **S** TUNEL staining of tumor tissues to assess apoptosis levels (*n* = 5). **T**-**V** Immunofluorescence analysis of differential expression of NRGN and β2m in tumor tissues (*n* = 5). **W** Flow cytometry analysis of CD8⁺ T cell activation status in different experimental groups (*n* = 5). **X** Flow cytometry analysis of tumor cell surface H-2Kb expression levels in different experimental groups (*n* = 5). *P < 0.05; **P < 0.01; ***P < 0.001; ****P < 0.0001; ns, not significant

To determine whether NRGN-mediated regulation of HCC growth depends on CD8⁺ T cells within the immune microenvironment, we employed a physiologically relevant murine orthotopic liver tumor model. CD8⁺ T cells were selectively depleted in vivo prior to tumor implantation (Fig. S2A), followed by orthotopic transplantation of murine Hep-53.4 cells with modulated Nrgn expression. Nrgn knockdown significantly inhibited tumor growth, as evidenced by markedly reduced tumor volume compared with control mice. However, in CD8⁺ T cell-depleted mice, the tumor-suppressive effect of Nrgn knockdown was markedly attenuated, and tumor volume was restored (Fig. 2A). Consistent with tumor growth kinetics, survival analysis showed that Nrgn knockdown significantly prolonged overall survival of tumor-bearing mice, whereas the survival benefit was largely abolished upon CD8⁺ T cell exhaustion (Fig. 2B). Systematic evaluation of tumor weight, tumor volume, liver weight/body weight ratio, and liver weight at the experimental endpoint mirrored the tumor growth and survival trends (Fig. 2C-F). These results indicate that the pro-tumorigenic effect of NRGN is at least partially dependent on CD8⁺ T cell-mediated antitumor immunity.

Having established the correlation between NRGN and CD8⁺ T cells, we next investigated the impact of NRGN on antigen presentation machinery at the tumor cell level. Overexpression of NRGN in Huh7 cells led to a marked reduction in MHC-I and β2M protein levels, as determined by Western blot (Fig. 2G, Fig. S2B). Conversely, NRGN knockdown in Hep3B cells resulted in a significant increase in MHC-I and β2M protein expression (Fig. 2H, Fig. S2C). Flow cytometric analysis further confirmed that NRGN overexpression decreased surface expression of MHC-I and β2M on Huh7 cells (Fig. 2I, Fig. S2D), whereas NRGN silencing in Hep3B cells enhanced their surface expression (Fig. 2J, Fig. S2E), consistent with the Western blot results. In murine Hep-53.4 cells, Nrgn overexpression significantly downregulated protein levels of H-2Kb (murine homolog of human MHC-I) and β2m (murine homolog of β2M) (Fig. 2K), whereas Nrgn knockdown produced the opposite effect, leading to increased expression of these molecules (Fig. 2L). Flow cytometry analysis corroborated these findings, showing that Nrgn overexpression decreased surface H-2Kb and β2m, while Nrgn silencing significantly enhanced their cell-surface expression (Fig. 2M and N; Fig. S2H, S2I).

To evaluate the impact of Nrgn modulation on HCC progression and the TIME in vivo, orthotopic liver tumors were established using Nrgn knockdown Hep-53.4 cells. Nrgn depletion significantly suppressed tumor growth and prolonged survival of tumor-bearing mice (Fig. 2O and P). At the experimental endpoint, tumor volume, tumor weight, liver weight/body weight ratio, and liver weight were all significantly reduced in the Nrgn knockdown group (Fig. 2Q and R; Fig. S2F, S2G). Histological analyses, including TUNEL staining (Fig. 2S) and Ki67 immunohistochemistry (Fig. S2J), revealed increased apoptosis and reduced proliferative activity in tumors upon Nrgn knockdown. Multiplex immunofluorescence analysis of Nrgn and β2m in tumor tissues showed that Nrgn knockdown led to markedly decreased Nrgn signal and significantly elevated β2m expression (Fig. 2T-V). Flow cytometric analysis of the TIME further demonstrated that Nrgn knockdown significantly enhanced functional activity of infiltrating CD8⁺ T cells (Fig. 2W). Concurrently, cell-surface expression of H-2Kb and β2m on tumor cells was markedly upregulated (Fig. 2X, Fig. S2K), consistent with the in vitro observations. Collectively, these findings indicate that NRGN promotes HCC progression at least partially by suppressing antigen presentation and impairing CD8⁺ T cell-mediated antitumor responses.

### Nrgn knockdown enhances CD8⁺ T cell-mediated antitumor immunity via H-2Kb-dependent antigen presentation

**Fig. 3:**
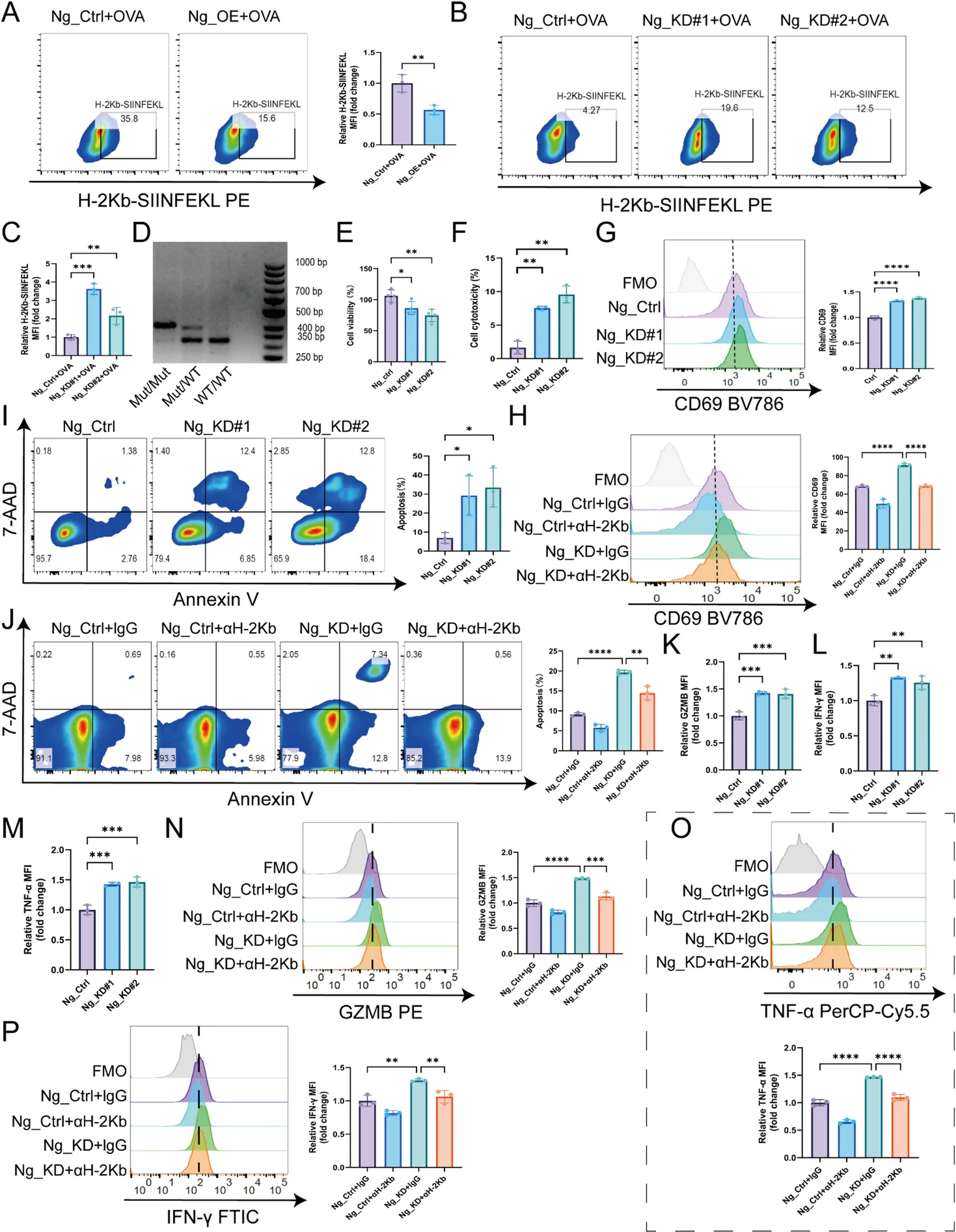
Nrgn regulates CD8⁺ T cell-mediated tumor cytotoxicity through MHC-I antigen presentation. **A** Flow cytometry analysis of antigen presentation capacity in Hep53.4_OVA cells following Nrgn overexpression (*n* = 3). **B**, **C** Flow cytometry analysis of antigen presentation capacity in Hep53.4_OVA cells after Nrgn knockdown (*n* = 3). **D** DNA gel electrophoresis confirming the homozygosity of OT-I mice. **E** OT-I mouse-derived CD8⁺ T cells were co-cultured with Nrgn-manipulated Hep53.4_OVA cells for 24 h, and tumor cell viability was assessed by CCK-8 assay (CD8⁺ T cell : tumor cell = 5:1) (*n* = 3). **F** LDH release assay to evaluate CD8⁺ T cell-mediated cytotoxicity after 24 h co-culture with Nrgn-manipulated Hep53.4_OVA cells (CD8⁺ T cell : tumor cell = 5:1) (*n* = 3). **G** Flow cytometry analysis of CD8⁺ T cell activation after 24 h co-culture with Nrgn-manipulated Hep53.4_OVA cells (CD8⁺ T cell : tumor cell = 5:1) (*n* = 3). **H** Flow cytometry analysis of CD8⁺ T cell activation in the presence of αH-2Kb blocking antibody during co-culture with Nrgn-manipulated Hep53.4_OVA cells (CD8⁺ T cell : tumor cell = 5:1) (*n* = 3). **I** Flow cytometry analysis of tumor cell apoptosis after 12 h co-culture with OT-I CD8⁺ T cells (CD8⁺ T cell : tumor cell = 5:1) (*n* = 3). **J** Flow cytometry analysis of tumor cell apoptosis in the presence of αH-2Kb blocking antibody during co-culture with OT-I CD8⁺ T cells (CD8⁺ T cell : tumor cell = 5:1) (*n* = 3). **K**-**M** Flow cytometry analysis of CD8⁺ T cell effector function markers after 24 h co-culture with Nrgn-manipulated Hep53.4_OVA cells (CD8⁺ T cell : tumor cell = 5:1) (*n* = 3). **N**-**P** Flow cytometry analysis of CD8⁺ T cell effector function markers in the presence of αH-2Kb blocking antibody during co-culture with Nrgn-manipulated Hep53.4_OVA cells (CD8⁺ T cell : tumor cell = 5:1) (*n* = 3). *P < 0.05; **P < 0.01; ***P < 0.001; ****P < 0.0001; ns, not significant

To clarify the impact of Nrgn modulation on CD8⁺ T cell cytotoxicity, we generated a stable murine Hep-53.4 cell line overexpressing ovalbumin (Hep-53.4_OVA). The OVA₍₂₅₇-₂₆₄₎ peptide (SIINFEKL) can be presented on the tumor cell surface via H-2Kb and specifically recognized by CD8⁺ T cells derived from OT-I transgenic mice. Nrgn was subsequently overexpressed or knocked down in Hep-53.4_OVA cells to assess its effect on antigen presentation and CD8⁺ T cell function. Flow cytometric analysis revealed that Nrgn overexpression significantly decreased surface SIINFEKL-H-2Kb complex levels (Fig. 3A), whereas Nrgn knockdown produced the opposite effect (Fig. 3B and C). OT-I mice were genotyped via DNA gel electrophoresis to confirm homozygosity, ensuring the accuracy of CD8⁺ T cell-based functional assays (Fig. 3D).

To determine whether Nrgn-mediated regulation of H-2Kb affects CD8⁺ T cell cytotoxicity, Hep-53.4_OVA tumor cells were co-cultured in direct contact with OT-I CD8⁺ T cells. CCK-8 assays demonstrated that tumor cell viability was significantly reduced under Nrgn knockdown conditions (Fig. 3E). Consistently, LDH release assays showed that Nrgn knockdown markedly enhanced CD8⁺ T cell-mediated cytotoxicity against tumor cells (Fig. 3F). Flow cytometric analysis further revealed that co-culture with Nrgn-knockdown tumor cells significantly increased CD8⁺ T cell activation (Fig. 3G). Notably, blocking the SIINFEKL peptide in the co-culture system substantially reversed the enhanced cytotoxic effect of CD8⁺ T cells (Fig. 3H).

Assessment of tumor cell apoptosis in the co-culture system indicated that Nrgn knockdown significantly increased tumor cell apoptosis (Fig. 3I), whereas blockade of the SIINFEKL peptide markedly abrogated this CD8⁺ T cell-mediated pro-apoptotic effect (Fig. 3J), suggesting dependence on H-2Kb-mediated antigen presentation. Further analysis of CD8⁺ T cell effector functions revealed that co-culture with Nrgn-knockdown tumor cells significantly upregulated the expression and secretion of GZMB, TNF-α, and IFN-γ compared with controls (Fig. 3K-M, Fig. S3A). In contrast, blocking the SIINFEKL peptide reversed the upregulation of these effector molecules (Fig. 3N-P). Collectively, these findings indicate that Nrgn knockdown enhances CD8⁺ T cell effector functions in vitro, and this effect is dependent on H-2Kb-mediated antigen presentation.

### β2m deficiency or H-2Kb blockade reverses Nrgn knockdown-mediated CD8⁺ T cell antitumor activity

**Fig. 4:**
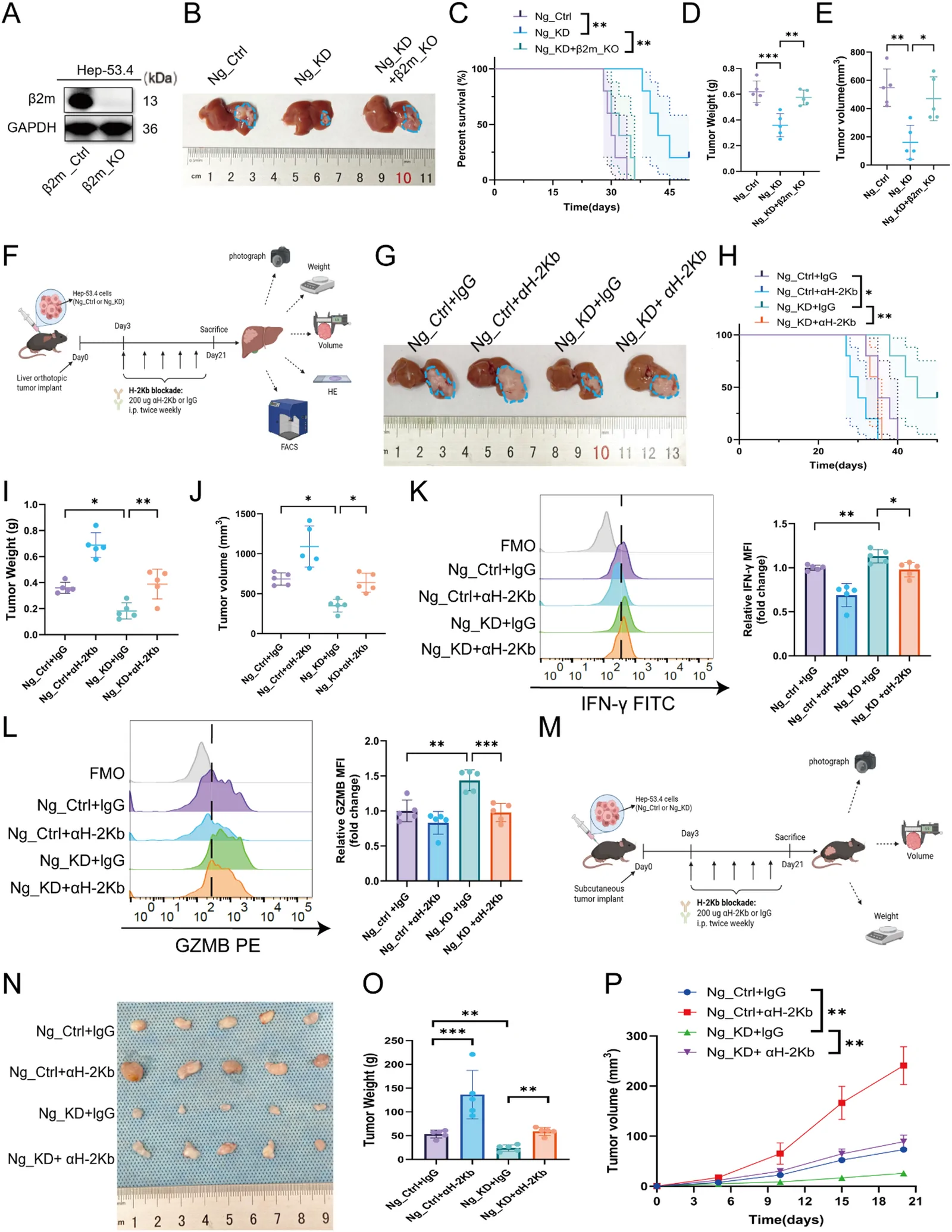
MHC-I antigen presentation is a critical mechanism underlying Nrgn-mediated immunosuppression in liver cancer. **A** Western blot analysis of β2m knockout efficiency in Hep53.4 cells (*n* = 3). **B** Representative images of orthotopic liver tumors in C57BL/6 mice established with Nrgn-knockdown or Nrgn-knockdown combined with β2m knockout Hep53.4 cells (*n* = 5). **C**-**E** Survival analysis (**C**), tumor weight (**D**), and tumor volume (**E**) of mice in the orthotopic liver cancer model. **F** Schematic illustration of the experimental design for orthotopic liver cancer model with Nrgn-modulated tumor cells combined with αH-2Kb antibody treatment. **G** Representative images of tumors in the orthotopic liver cancer model (*n* = 5). **H**-**J** Survival analysis (**H**), tumor weight (**I**), and tumor volume (**J**) under combined αH-2Kb treatment. **K**, **L** Flow cytometry analysis of CD8⁺ T cell effector status in the TIME of C57BL/6 mice after Nrgn modulation combined with αH-2Kb treatment. **M** Schematic illustration of the experimental workflow for subcutaneous tumor model in C57BL/6 mice using Nrgn-modulated tumor cells combined with αH-2Kb treatment. **N** Representative images of subcutaneous tumors (*n* = 5). **O**, **P** Tumor volume (**O**) and final tumor weight (**P**) in the subcutaneous tumor model. *P < 0.05; **P < 0.01; ***P < 0.001; ****P < 0.0001; ns, not significant

To further determine whether Nrgn expression in tumor cells regulates CD8⁺ T cell activation via the antigen presentation pathway, β2m was first knocked out in Hep-53.4 cells (Fig. 4A). β2m is an essential component of H-2Kb molecules, and its loss significantly impairs the stability and function of H-2Kb [29]. Nrgn was then knocked down in these β2m-deficient cells, and murine orthotopic liver tumor models were established (Fig. 4B). Results showed that Nrgn knockdown significantly inhibited tumor growth compared with controls, as indicated by reduced tumor volume, prolonged survival, and decreased tumor weight, liver-to-body weight ratio, and liver weight. However, these antitumor effects were partially reversed in the β2m-deficient background (Fig. 4C-E, Fig. S3B, S3C), indicating that Nrgn-mediated regulation of tumor progression via CD8⁺ T cells is at least partially dependent on β2m-dependent MHC-I antigen presentation.

To further validate the critical role of H-2Kb in Nrgn-mediated antitumor immunity, Hep-53.4 cells with Nrgn knockdown were used to establish orthotopic liver tumors in mice, followed by in vivo blockade with αH-2Kb antibody (Fig. 4F and G). Nrgn knockdown significantly reduced tumor volume and prolonged survival, while also decreasing tumor weight, liver-to-body weight ratio, and liver weight. However, these antitumor effects were markedly reversed upon H-2Kb blockade (Fig. 4H-J; Fig. S3D, S3E). During the experiment, mouse body weight was continuously monitored (Fig. S3F), and histological analysis of heart, liver, spleen, lung, and kidney by H&E staining revealed no significant tissue damage (Fig. S3G), indicating that in vivo H-2Kb blockade was well tolerated under these experimental conditions. Flow cytometric analysis of the TIME demonstrated that the Nrgn knockdown-mediated enhancement of CD8⁺ T cell effector function was significantly abrogated by αH-2Kb antibody treatment (Fig. 4K and L). These findings were further corroborated in a subcutaneous tumor model (Fig. 4M). Nrgn knockdown-induced suppression of tumor growth was similarly reversed by αH-2Kb antibody treatment, as evidenced by increased tumor volume and tumor burden compared with the control group (Fig. 4N-P).

### NRGN regulates tumor antigen presentation and CD8⁺ T cell-mediated antitumor immunity via IRF8

**Fig. 5:**
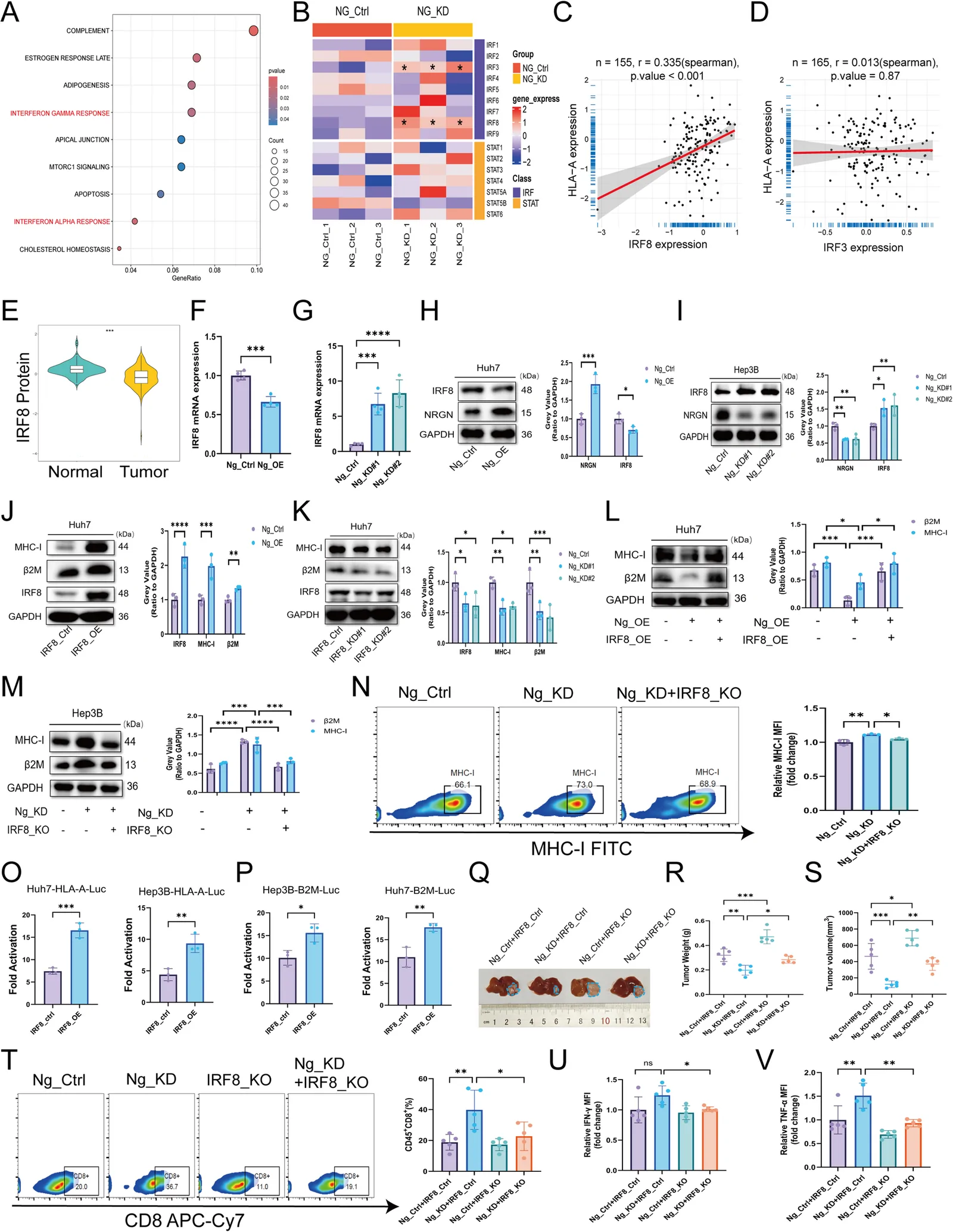
NRGN suppresses MHC-I expression and impairs CD8⁺ T cell-mediated antitumor immunity via downregulation of IRF8. **A** Functional enrichment analysis of differentially expressed genes between control and NRGN-knockdown Hep3B cells. **B** Heatmap showing expression of IRF and STAT family-related genes in the two groups. **C**, **D** Correlation analysis between IRF8 (**C**) or IRF3 (**D**) protein expression and HLA-A protein expression based on CPTAC database. **E** Differential expression of IRF8 protein between liver cancer tissues and adjacent normal tissues analyzed using CPTAC database. **F**, **G** qPCR analysis of IRF8 mRNA levels in Huh7 (**F**) and Hep3B (**G**) cells following NRGN overexpression (*n* = 3). **H**, **I** Western blot analysis of IRF8 protein expression in Huh7 cells overexpressing NRGN (**H**) and in Hep3B cells with NRGN knockdown (**I**) (*n* = 3). **J**, **K** Western blot analysis of MHC-I and β2M protein levels following IRF8 overexpression (**J**) or knockdown (**K**) in Huh7 cells (*n* = 3). **L** Western blot analysis of MHC-I and β2M protein levels in Huh7 cells overexpressing NRGN with IRF8 rescue (*n* = 3). **M** Western blot analysis of MHC-I and β2M protein levels in Hep3B cells with NRGN knockdown and IRF8 rescue (*n* = 3). **N** Flow cytometry analysis of cell surface MHC-I expression in Hep3B cells with NRGN knockdown and IRF8 rescue (*n* = 3). **O** Dual-luciferase reporter assay showing the effect of IRF8 on HLA-A promoter transcriptional activity in Huh7 and Hep3B cells (*n* = 3). **P** Dual-luciferase reporter assay showing the effect of IRF8 on B2M promoter transcriptional activity in Huh7 and Hep3B cells (*n* = 3). **Q** Representative images of orthotopic liver tumors in C57BL/6 mice established with Nrgn-modulated Hep53.4 cells combined with IRF8 knockout (*n* = 5). **R**, **S** Tumor weight (**R**) and tumor volume (**S**) in the orthotopic liver cancer model. **T** Flow cytometry analysis of CD8⁺ T cell proportion in the TME across four groups (*n* = 5). **U**, **V** Flow cytometry analysis of intracellular IFN-γ (**U**) and TNF-α (**V**) mean fluorescence intensity (MFI) in CD8⁺ T cells in the TME across four groups (*n* = 5). *P < 0.05; **P < 0.01; ***P < 0.001; ****P < 0.0001; ns, not significant

To investigate the potential molecular mechanism by which Nrgn regulates tumor antigen presentation, differential gene expression analysis was performed on the transcriptomic data of NRGN control and knockdown Hep3B cells, followed by KEGG pathway enrichment analysis. The results revealed that the differentially expressed genes were mainly enriched in interferon-related signaling pathways (Fig. 5A), suggesting that NRGN may influence antigen presentation through the interferon signaling axis. Further analysis of representative IRF and STAT family genes within the interferon pathway by heatmap showed that IRF3 and IRF8 were significantly upregulated in NRGN knockdown cells, with IRF8 displaying a more pronounced fold change (Fig. 5B; Fig. S4A, S4B). Based on these findings, the CPTAC database was used to evaluate correlations between IRF8 or IRF3 protein levels and antigen presentation-related proteins, including HLA-A, HLA-B, HLA-C, and B2M. Only IRF8 protein levels were significantly positively correlated with all four antigen presentation molecules (Fig. 5C and D; Fig. S4D-S4I), guiding subsequent studies to focus on IRF8. Consistently, TCGA data analysis revealed that IRF8 mRNA was significantly downregulated in tumor tissues compared with normal tissues (Fig. S4C), and CPTAC proteomic analysis further confirmed decreased IRF8 protein expression in tumors (Fig. 5E).

In vitro experiments demonstrated that NRGN overexpression in Huh7 cells significantly downregulated IRF8 mRNA levels, whereas NRGN knockdown in Hep3B cells markedly increased IRF8 mRNA expression (Fig. 5F and G). Western blot analysis confirmed these observations at the protein level, with NRGN overexpression suppressing IRF8 protein and NRGN knockdown enhancing its expression (Fig. 5H and I). Next, the role of IRF8 in regulating antigen presentation-related molecules was assessed. Overexpression or knockdown of IRF8 in Huh7 cells revealed that IRF8 overexpression significantly increased MHC-I and β2M protein levels, whereas IRF8 knockdown suppressed their expression (Fig. 5J and K). To determine whether NRGN regulation of antigen presentation depends on interferon signaling, cells under various treatment conditions were analyzed. Western blot results showed that NRGN overexpression reduced IRF8, MHC-I, and β2M protein levels, which were partially restored upon exogenous interferon treatment. Conversely, NRGN knockdown upregulated IRF8, MHC-I, and β2M protein levels, and further interferon stimulation enhanced this upregulation (Fig. S5J, S5K).

To further clarify whether NRGN regulation of MHC-I and β2M is IRF8-dependent, rescue experiments were performed in Huh7 cells by simultaneous overexpression of NRGN and IRF8. Western blot analysis showed that IRF8 restoration reversed the suppression of MHC-I and β2M protein levels induced by NRGN overexpression (Fig. 5L). Similar results were obtained in Hep3B cells: NRGN knockdown increased MHC-I and β2M expression, while additional IRF8 knockout in the NRGN knockdown background reversed this upregulation (Fig. 5M). These findings indicate that NRGN-mediated regulation of MHC-I and β2M largely depends on IRF8. Flow cytometric analysis of tumor cell surface antigen presentation molecules further confirmed these observations. In both Hep3B (Fig. 5N, Fig. S4M) and Huh7 cells (Fig. S4L), the surface expression changes of MHC-I and β2M under NRGN manipulation with IRF8 rescue were consistent with the Western blot results, further supporting a critical role for IRF8 in NRGN-mediated antigen presentation regulation. Subsequently, Western blot analysis of clinical samples from 12 HCC patients revealed that NRGN protein expression was negatively correlated with IRF8 and MHC-I molecule levels (Fig. S5A). Luciferase reporter assays demonstrated that IRF8 directly binds to the promoters of HLA-A (Fig. 5O) and B2M (Fig. 5P), significantly enhancing their transcriptional activity, indicating that IRF8 acts as a transcription factor to directly promote MHC-I and β2M expression.

To validate the role of IRF8 in Nrgn-mediated tumor regulation in vivo, IRF8 was knocked out in mouse Hep53.4 cells (Fig. S5B), followed by Nrgn knockdown and establishment of orthotopic liver tumor models (Fig. 5Q). Nrgn knockdown-mediated tumor growth inhibition was markedly reversed upon IRF8 knockout, as evidenced by increased tumor volume, tumor weight, liver-to-body weight ratio, and liver weight (Fig. 5R and S; Fig. S5C, S5D). Correspondingly, the survival benefit of Nrgn knockdown was significantly diminished in IRF8-deficient mice (Fig. S5E). Flow cytometric analysis of the TIME revealed that Nrgn knockdown significantly increased CD8⁺ T cell infiltration, whereas IRF8 deletion substantially reduced this effect (Fig. 5T). Similar trends were observed for IFN-γ⁺CD8⁺ and TNF-α⁺CD8⁺ T cell populations (Fig. 5U and V; Fig. S5F, S5G). Together, these results demonstrate that IRF8 deficiency significantly attenuates Nrgn-mediated enhancement of CD8⁺ T cell-dependent antitumor immunity in orthotopic liver tumors, confirming the critical role of IRF8 in NRGN-mediated regulation of the TIME in vivo.

### NRGN regulates IRF8 mRNA stability via FMR1

**Fig. 6:**
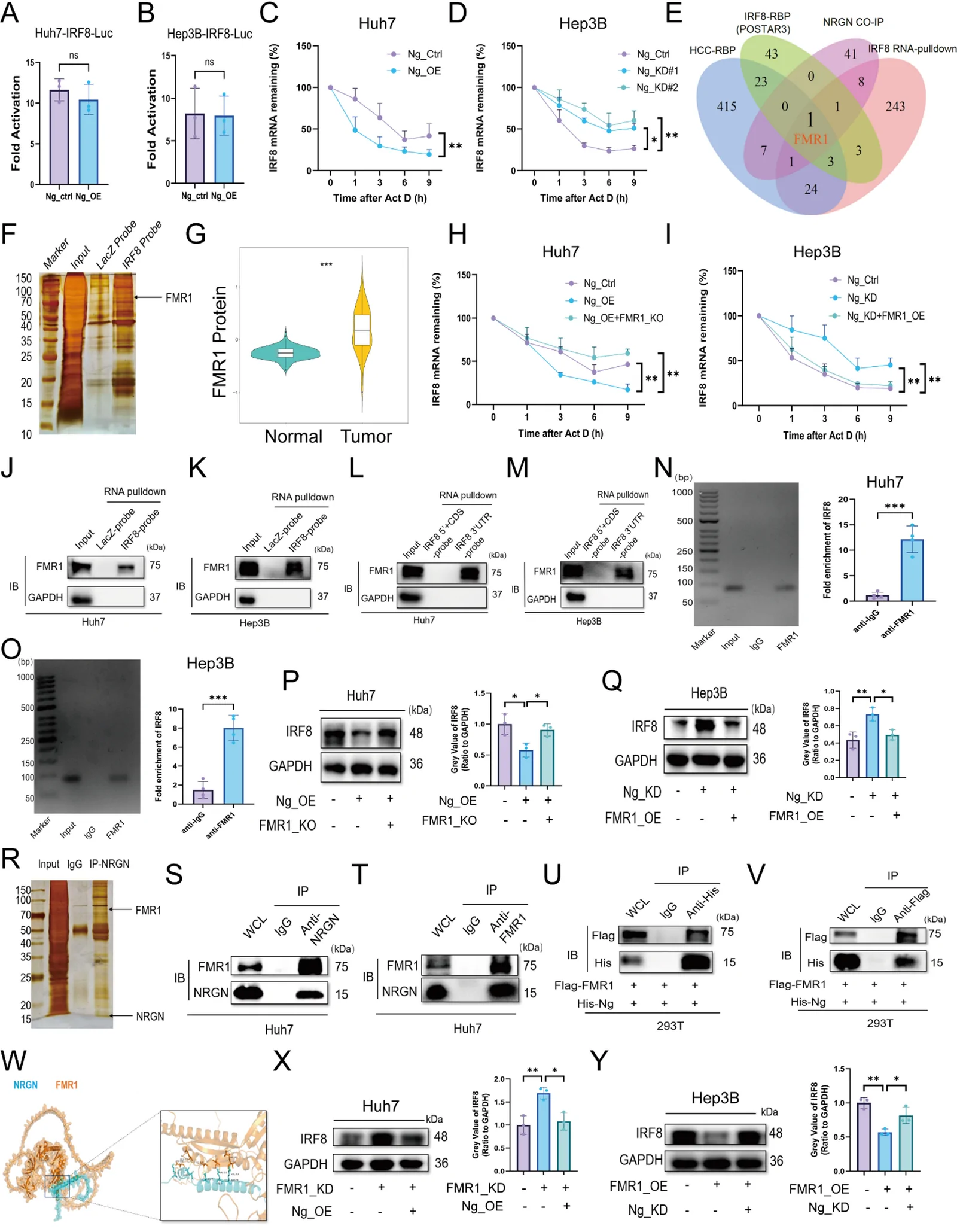
NRGN downregulates IRF8 expression by interacting with FMR1 to reduce IRF8 mRNA stability. **A**, **B** Dual-luciferase reporter assay showing the effect of NRGN on IRF8 promoter transcriptional activity in Huh7 (**A**) and Hep3B (**B**) cells (*n* = 3). **C**, **D** Actinomycin D assay analyzing the regulatory effect of NRGN on IRF8 mRNA stability in Huh7 (**C**) and Hep3B (**D**) cells (*n* = 4). **E** Venn diagram showing the intersection of proteins identified by IRF8 RNA pull-down, NRGN Co-IP interactome, IRF8-binding proteins predicted by POSTAR3, and prognostic RNA-binding proteins in liver cancer. **F** Silver staining of IRF8 RNA pull-down assay. **G** Differential expression of FMR1 protein in liver cancer tissues and adjacent normal tissues based on CPTAC database. **H**, **I** Actinomycin D assay evaluating the effect of NRGN intervention combined with FMR1 rescue on IRF8 mRNA stability in Huh7 (**H**) and Hep3B (**I**) cells (*n* = 4). **J**, **K** RNA pull-down assay detecting the binding of IRF8 mRNA to FMR1 protein in Huh7 (**J**) and Hep3B (**K**) cells. **L**, **M** RNA pull-down assay detecting the binding between FMR1 protein and different truncated fragments of IRF8 mRNA in Huh7 (**L**) and Hep3B (**M**) cells. **N**, **O** RIP-qPCR validating the interaction between FMR1 protein and IRF8 mRNA in Huh7 (**N**) and Hep3B (**O**) cells (*n* = 3). **P**, **Q** Western blot analysis of IRF8 protein expression in Huh7 (**P**) and Hep3B (**Q**) cells following NRGN intervention and FMR1 rescue (*n* = 3). **R** Silver staining of NRGN Co-IP samples. **S**, **T** Co-IP detecting endogenous interaction between NRGN and FMR1. **U**, **V** Co-IP detecting exogenous interaction between NRGN and FMR1. **W** AlphaFold-based structural prediction model of NRGN-FMR1 protein interaction. **X**, **Y** Western blot analysis in Huh7 cells showing the effect of NRGN rescue on IRF8 protein levels after FMR1 knockout (**X**) or overexpression (**Y**) (*n* = 3). *P < 0.05; **P < 0.01; ***P < 0.001; ****P < 0.0001; ns, not significant

We next explored the molecular mechanism by which NRGN regulates IRF8 expression. Luciferase reporter assays analyzing IRF8 promoter activity showed that NRGN overexpression did not significantly affect IRF8 transcriptional activity (Fig. 6A and B), suggesting that NRGN does not regulate IRF8 at the transcriptional level. Considering this, we investigated post-transcriptional regulation. Actinomycin D treatment was applied to block nascent transcription, and results demonstrated that NRGN overexpression in Huh7 cells significantly decreased IRF8 mRNA stability (Fig. 6C), whereas NRGN knockdown in Hep3B cells markedly enhanced IRF8 mRNA stability (Fig. 6D), indicating that NRGN may influence IRF8 expression by modulating mRNA stability.

Based on these findings, we hypothesized that NRGN might indirectly regulate IRF8 stability via interacting RNA-binding proteins. To test this, IRF8 RNA pulldown and NRGN co-immunoprecipitation (Co-IP) experiments were performed, followed by mass spectrometry analysis of the associated proteins. These results were intersected with IRF8-binding proteins predicted by the POSTAR3 database and previously reported RNA-binding proteins associated with HCC prognosis, ultimately identifying FMR1 as a common candidate (Fig. 6E and F; Fig. S5K). TCGA analysis revealed that FMR1 mRNA was significantly upregulated in tumor tissues (Fig. S5H), and CPTAC data confirmed increased FMR1 protein expression in tumors (Fig. 6G) with a negative correlation to IRF8 protein (Fig. S5I). To verify the role of FMR1 in NRGN-mediated regulation of IRF8 stability, NRGN was overexpressed in Huh7 cells with simultaneous FMR1 knockout (Fig. S5J). Actinomycin D treatment followed by qPCR analysis revealed that FMR1 deletion significantly rescued the decreased IRF8 mRNA stability caused by NRGN overexpression (Fig. 6H). Similarly, in Hep3B cells, NRGN knockdown enhanced IRF8 stability, whereas concurrent FMR1 overexpression significantly attenuated this effect (Fig. 6I), indicating that NRGN-mediated regulation of IRF8 stability is FMR1-dependent, Subsequent investigations further revealed that NRGN was unable to independently regulate IRF8 mRNA stability in the absence of FMR1 (Figs. S6A and S6B). To investigate the in vivo role of FMR1 in NRGN-mediated tumor regulation, FMR1 was overexpressed in murine Hep53.4 cells, followed by Nrgn knockdown, and an orthotopic liver tumor model was subsequently established (Fig. S6C). The results showed that, under conditions of FMR1 overexpression, the tumor growth inhibition induced by Nrgn knockdown was significantly reversed, as evidenced by increased tumor weight, tumor volume, and liver-to-body weight ratio (Figs. S6D-S6F). Flow cytometric analysis of the TIME, further revealed that Nrgn knockdown markedly enhanced the infiltration of effector CD8⁺ T cells, whereas FMR1 overexpression attenuated this effect (Figs. S6G and S6H). In contrast, no significant changes were observed in the proportions of CD4⁺ T cells or NK cells within the tumor microenvironment (Figs. S6I, S6J). These findings demonstrate that FMR1 overexpression significantly impairs the enhancement of NRGN-mediated CD8⁺ T cell-dependent antitumor immune responses in orthotopic liver tumors, thereby suggesting that FMR1 contributes to the regulation of the in vivo TIME in the context of NRGN signaling.

RNA pulldown assays further confirmed the direct binding of FMR1 to IRF8 mRNA. Using full-length IRF8 probes, FMR1 was shown to bind IRF8 mRNA (Fig. 6J and K). Truncated IRF8 probes identified the 3′UTR region as the primary FMR1 binding site (Fig. 6L and M). RIP assays in Huh7 (Fig. 6N) and Hep3B (Fig. 6O) cells further verified specific FMR1-IRF8 mRNA interactions. At the protein level, Western blot analysis showed that FMR1 knockout significantly attenuated NRGN-mediated suppression of IRF8 protein in Huh7 cells (Fig. 6P), with consistent results obtained in Hep3B cells (Fig. 6Q), confirming the critical mediatory role of FMR1 in NRGN-regulated IRF8 stability. Co-IP experiments demonstrated the interaction between NRGN and FMR1 (Fig. 6R). Endogenous Co-IP in Huh7 cells revealed that NRGN and FMR1 physically interact at the native level (Fig. 6S and T). Consistently, this interaction was confirmed in human HCC tissues (Fig. S7A), but was not detected in matched normal liver tissues (Fig. S7B). Exogenous Co-IP experiments in 293T cells using differentially tagged NRGN and FMR1 constructs confirmed a stable interaction between the two proteins (Fig. 6U and V). AlphaFold-based structural modeling predicted potential binding interfaces between NRGN and FMR1 (Fig. 6W), providing computational support for their direct interaction. Finally, we assessed whether NRGN is required for FMR1-mediated regulation of IRF8. In Huh7 cells, FMR1 was first knocked down, followed by NRGN rescue. Western blot analysis showed that FMR1-mediated regulation of IRF8 partially depends on NRGN (Fig. 6X). Similar results were observed in Hep3B cells (Fig. 6Y), further indicating that NRGN participates in and regulates FMR1-mediated control of IRF8 mRNA stability.

### NRGN regulates FMR1 protein stability by inhibiting FMR1-MYCBP2 complex formation

**Fig. 7:**
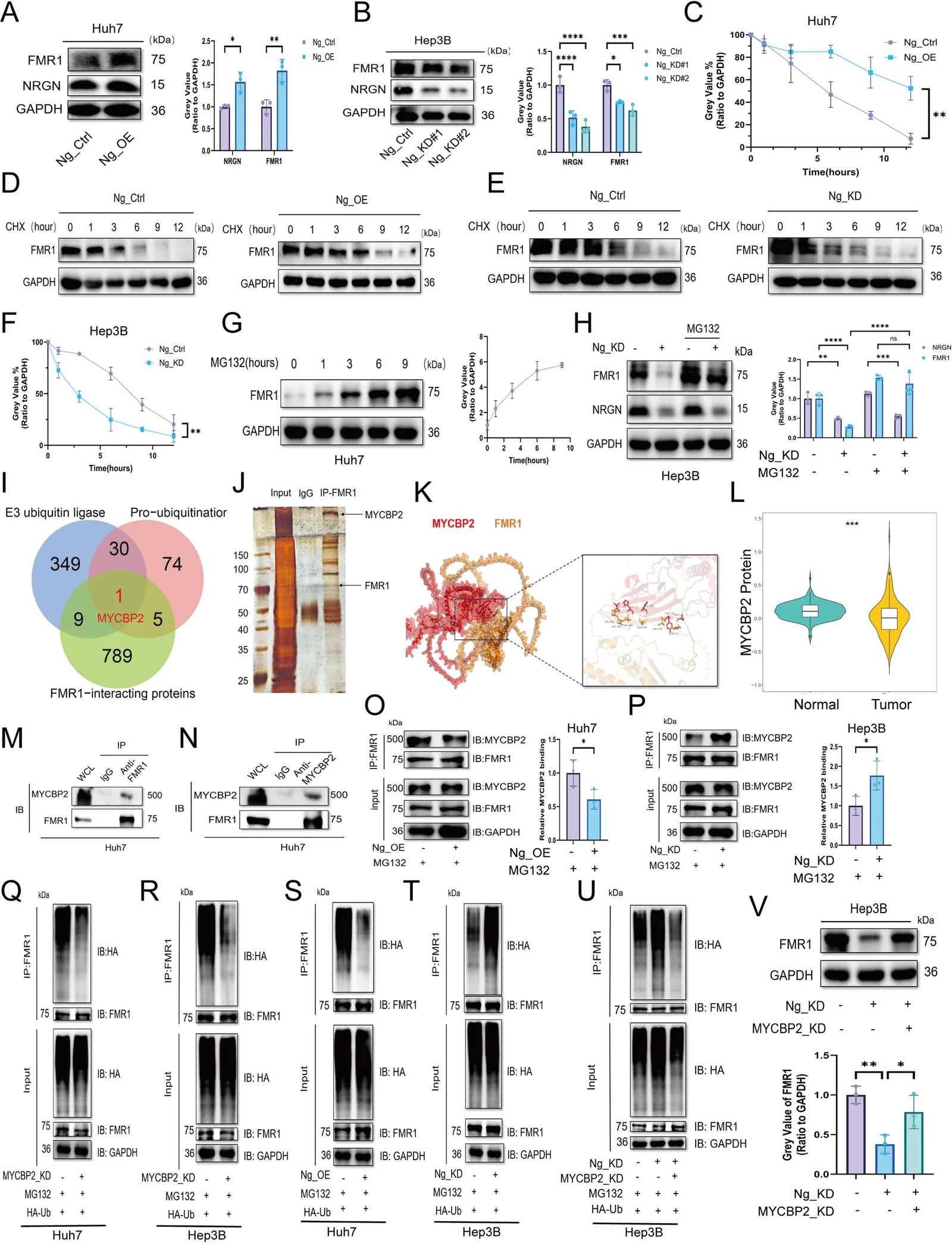
NRGN enhances FMR1 protein stability by inhibiting MYCBP2-mediated ubiquitination. **A**, **B** Western blot analysis showing FMR1 protein expression levels in Huh7 (**A**) and Hep3B (**B**) cells following NRGN intervention (*n* = 3). **C**, **D** Western blot analysis of FMR1 protein expression in Huh7 cells after NRGN overexpression and cycloheximide (CHX) treatment (*n* = 3). **E**, **F** Western blot analysis of FMR1 protein expression in Hep3B cells after NRGN knockdown and CHX treatment (*n* = 3). **G** Western blot analysis of FMR1 protein expression in Huh7 cells following treatment with the proteasome inhibitor MG132 (50 μg/mL) (*n* = 3). **H** Western blot analysis of FMR1 protein levels in NRGN-knockdown and control cells under MG132 treatment to further assess the role of NRGN in FMR1 protein degradation (*n* = 3). **I** Venn diagram showing the intersection of FMR1 Co-IP-identified interactors, ubiquitination-related proteins enriched in GSEA analysis, and literature-reported E3 ubiquitin ligases. **J** Silver staining of FMR1 Co-IP samples. **K** AlphaFold-based structural prediction model of FMR1-MYCBP2 protein interaction. **L** Differential expression of MYCBP2 protein in liver cancer tissues and adjacent normal tissues based on CPTAC database. **M**, **N** Co-IP experiments validating the interaction between FMR1 and MYCBP2 in Huh7 cells. **O**, **P** Co-IP analysis of FMR1-MYCBP2 binding in Huh7 (**O**) and Hep3B (**P**) cells following NRGN intervention (*n* = 3). **Q**, **R** Western blot analysis of FMR1 ubiquitination levels in Huh7 (**Q**) and Hep3B (**R**) cells after MYCBP2 knockdown (*n* = 3). **S**, **T** Western blot analysis of FMR1 ubiquitination levels in Huh7 (**S**) and Hep3B (**T**) cells following NRGN intervention (*n* = 3). **U** Western blot analysis of FMR1 ubiquitination levels in Hep3B cells with NRGN knockdown combined with MYCBP2 rescue (*n* = 3). **V** Western blot analysis of FMR1 protein expression in Hep3B cells with NRGN knockdown combined with MYCBP2 rescue (*n* = 3). *P < 0.05; **P < 0.01; ***P < 0.001; ****P < 0.0001; ns, not significant

Given our previous findings that NRGN regulates IRF8 mRNA stability via FMR1, we further investigated the molecular mechanism by which NRGN modulates FMR1 levels and its subsequent effect on IRF8 regulation. Western blot analysis revealed that NRGN overexpression in Huh7 cells significantly upregulated FMR1 protein levels (Fig. 7A), whereas NRGN knockdown in Hep3B cells led to a marked decrease in FMR1 protein (Fig. 7B), indicating that NRGN expression is closely associated with FMR1 protein levels. To determine whether NRGN affects FMR1 via protein stability, cycloheximide (CHX) was used to block de novo protein synthesis. Results showed that FMR1 gradually degraded over time, whereas NRGN overexpression significantly delayed FMR1 degradation (Fig. 7C and D). Conversely, NRGN knockdown accelerated FMR1 degradation (Fig. 7E and F), suggesting that NRGN plays a critical role in maintaining FMR1 protein stability. Considering the key role of the ubiquitin-proteasome system in protein degradation, we treated HCC cells with the proteasome inhibitor MG132. MG132 treatment markedly inhibited FMR1 degradation, leading to intracellular accumulation (Fig. 7G), indicating that FMR1 degradation is primarily proteasome-dependent. Notably, in the context of NRGN knockdown, the decreased FMR1 protein levels were significantly rescued by MG132 (Fig. 7H), further supporting that NRGN regulates FMR1 stability via the ubiquitin-proteasome pathway.

Although NRGN itself lacks structural or functional features of E3 ubiquitin ligases or deubiquitinases, previous studies have confirmed a direct interaction between NRGN and FMR1. We therefore hypothesized that NRGN might indirectly regulate FMR1 ubiquitination and stability by modulating FMR1 interactions with its associated ubiquitin ligases. Based on prior FMR1 Co-IP experiments, the immunoprecipitated complex was subjected to mass spectrometry. By intersecting FMR1-interacting proteins with literature-reported E3 ubiquitin ligases and “positive regulation of ubiquitination” gene sets from GSEA, MYCBP2 was identified as a candidate E3 ligase (Fig. 7I and J; Fig. S7E). AlphaFold structural predictions also indicated a potential interaction interface between FMR1 and MYCBP2 (Fig. 7K). CPTAC database analysis revealed that MYCBP2 protein expression is significantly decreased in tumor tissues (Fig. 7L). Endogenous Co-IP in Huh7 cells further confirmed a specific interaction between FMR1 and MYCBP2 (Fig. 7M and N). Consistently, this interaction was confirmed in human HCC tissues (Fig. S7C), but was not detected in matched normal liver tissues (Fig. S7D). Since NRGN directly interacts with FMR1, we next investigated whether NRGN regulates FMR1 ubiquitination by modulating FMR1-MYCBP2 complex formation. Co-IP and Western blot analysis in Huh7 cells overexpressing NRGN combined with MG132 treatment showed that NRGN overexpression markedly reduced FMR1-MYCBP2 binding (Fig. 7O). Conversely, NRGN knockdown in Hep3B cells enhanced the interaction between FMR1 and MYCBP2 (Fig. 7P).

To assess the role of MYCBP2 in FMR1 ubiquitination, MYCBP2 knockdown efficiency was first verified (Fig. S7F, S7G). MYCBP2 knockdown in Huh7 and Hep3B cells significantly decreased FMR1 ubiquitination (Fig. 7Q and R), indicating that MYCBP2 serves as a key E3 ligase mediating FMR1 ubiquitination. Further analysis of NRGN regulation of MYCBP2-mediated FMR1 ubiquitination showed that NRGN overexpression significantly suppressed FMR1 ubiquitination (Fig. 7S), whereas NRGN knockdown promoted FMR1 ubiquitination (Fig. 7T). Notably, rescue experiments demonstrated that the increased FMR1 ubiquitination induced by NRGN knockdown could be reversed by concurrent MYCBP2 knockdown (Fig. 7U). Correspondingly, the decreased FMR1 protein levels caused by NRGN knockdown were also rescued by MYCBP2 knockdown (Fig. 7V).

### PD-1 antibody combined with NRGN intervention significantly enhances CD8⁺ T cell-mediated antitumor immunity in HCC

**Fig. 8:**
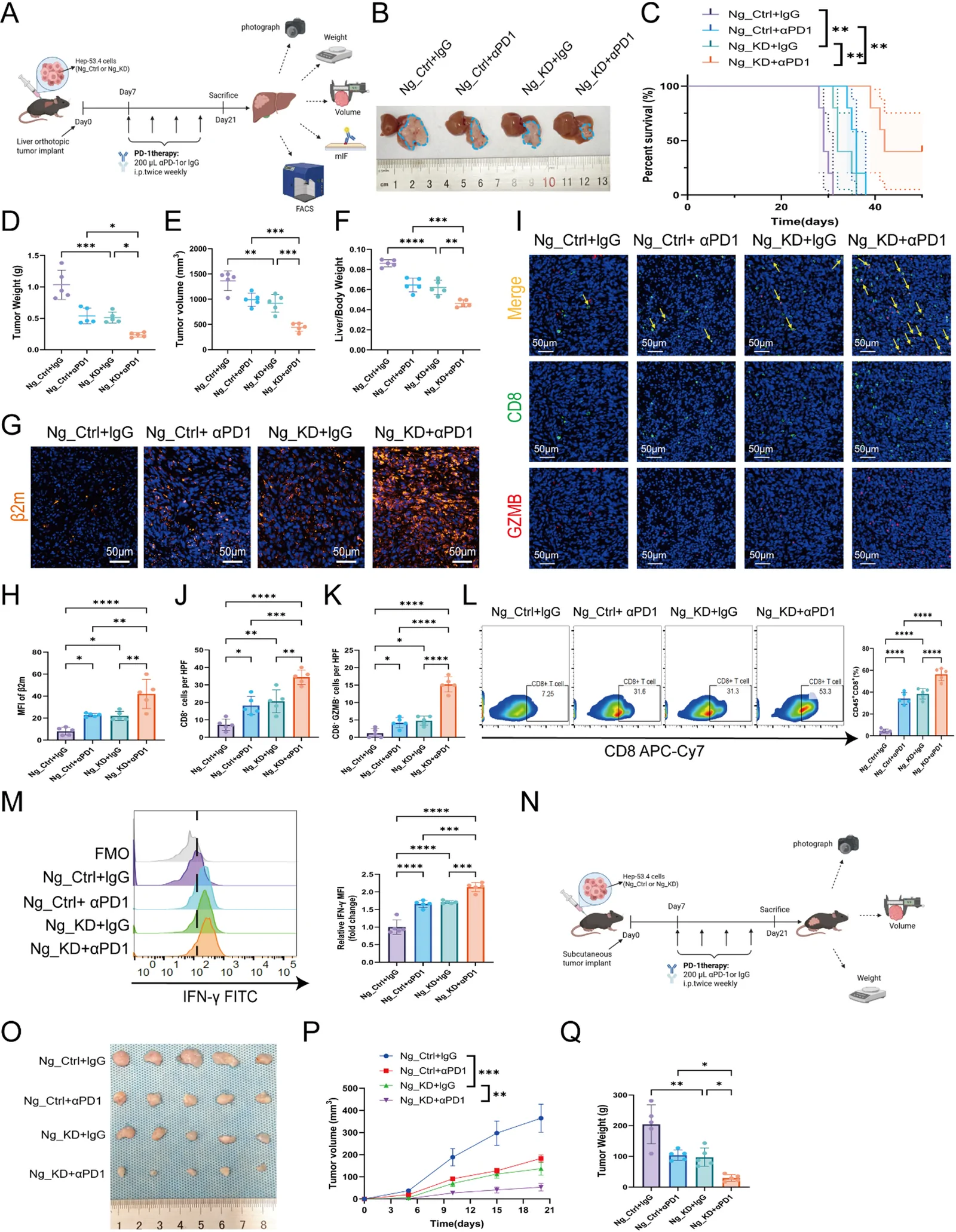
NRGN intervention combined with PD-1 antibody enhances CD8⁺ T cell-mediated antitumor immunity in HCC. **A** Schematic diagram of the experimental workflow for constructing orthotopic liver tumors in C57BL/6 mice using NRGN-intervened tumor cells and treating with αPD-1 antibody. **B** Representative images of orthotopic liver tumors (*n* = 5). **C**-**F** Survival analysis (**C**), tumor weight (**D**), tumor volume (**E**), and liver weight/body weight ratio (**F**) of mice bearing orthotopic liver tumors. **G**, **H** Immunofluorescence analysis of β2M protein expression in tumors from the four treatment groups (*n* = 5). **I**-**K** Multiplex immunofluorescence analysis of CD8⁺ T cell infiltration and effector CD8⁺ T cell distribution in tumors from the four treatment groups (*n* = 5). **L** Flow cytometry analysis of the proportion of CD8⁺ T cells in the TIME of the four groups (*n* = 5). **M** Flow cytometry analysis of intracellular IFN-γ MFI in CD8⁺ T cells in the TME across four groups (*n* = 5). **N** Schematic diagram of the experimental workflow for constructing subcutaneous tumors in C57BL/6 mice using NRGN-intervened tumor cells combined with αPD-1 treatment. **O** Representative images of subcutaneous tumors (*n* = 5). **P**, **Q** Tumor volume changes (**P**) and terminal tumor weight (**Q**) in the subcutaneous tumor model. *P < 0.05; **P < 0.01; ***P < 0.001; ****P < 0.0001; ns, not significant

To further evaluate the effect of PD-1 antibody combined with NRGN intervention on CD8⁺ T cell-mediated antitumor function in the TIME of HCC, we established an orthotopic mouse HCC model using Hep-53.4 cells with NRGN knockdown (Fig. 8A and B). Survival analysis showed that NRGN knockdown significantly prolonged the survival of tumor-bearing mice compared with the control group (Fig. 8C). Consistently, NRGN knockdown markedly suppressed tumor growth, as evidenced by reductions in tumor volume, tumor weight, and tumor weight/body weight ratio. Upon additional in vivo PD-1 antibody treatment, these antitumor effects were further enhanced, suggesting that NRGN intervention can significantly improve the therapeutic efficacy of PD-1 blockade (Fig. 8D-F; Fig. S7J). During the experiments, continuous monitoring of mouse body weight showed stable changes with no significant weight loss observed across groups (Fig. S7K). Furthermore, H&E staining of major organs (heart, liver, spleen, lung, and kidney) at the experimental endpoint revealed no obvious histopathological abnormalities, indicating that combined PD-1 antibody and NRGN intervention is well tolerated under the experimental conditions (Fig. S7L).

Next, to systematically assess the impact of NRGN knockdown combined with PD-1 antibody treatment on CD8⁺ T cell infiltration and functional status within the TIME, tumor tissues from the four treatment groups were subjected to immunofluorescence and flow cytometry analyses. Immunofluorescence staining for β2m showed that NRGN knockdown significantly upregulated β2m expression, and this effect was further enhanced upon PD-1 antibody treatment (Fig. 8G and H). Concurrently, multiplex immunofluorescence analysis revealed that, compared with the control group, NRGN knockdown markedly increased CD8⁺ T cell infiltration and activation within tumor tissues, which was further potentiated by in vivo PD-1 antibody administration (Fig. 8I and K). Flow cytometry analysis corroborated these findings, demonstrating that PD-1 antibody treatment synergized with NRGN knockdown to significantly enhance both CD8⁺ T cell infiltration and effector function in the TME (Fig. 8L and M; Fig. S7H, S7I). To verify the consistency of this effect across different in vivo models, a subcutaneous mouse tumor model was generated using Hep-53.4 cells with NRGN knockdown (Fig. 8N). Similarly, the results showed that anti–PD-1 antibody treatment synergistically enhanced the reduction in tumor volume and weight induced by NRGN knockout (Fig. 8O-Q). Taken together, these findings demonstrate that PD-1 blockade enhances NRGN-mediated CD8⁺ T cell–dependent antitumor immunity in HCC.

## Discussion

Tumor immune evasion is a central barrier limiting the efficacy of immunotherapy in HCC, with defects in antigen presentation recognized as a key mechanism. Tumor cells evade immune surveillance by downregulating MHC-I molecules and their essential components, thereby impairing the presentation of tumor antigens to CD8⁺ T cells [15, 30]. Previous studies have indicated that impaired antigen presentation in HCC may underlie the low response rates to immune checkpoint inhibitors [8]. However, how endogenous tumor molecules systematically regulate antigen presentation and, in turn, modulate CD8⁺ T cell function remains largely unexplored. In this study, extending beyond the traditional neurobiological context in which NRGN has predominantly been investigated, we explored its previously uncharacterized role in HCC and systematically delineated its critical contribution to antigen presentation defects and immune evasion at the molecular, phenotypic, and functional levels.

Transcriptomic analysis revealed that Nrgn knockdown significantly enriched pathways related to MHC-I-mediated antigen processing and presentation, with multiple key antigen-presentation genes consistently upregulated. In vitro experiments further validated that NRGN suppresses the expression of MHC-I and β2M and reduces their stable surface presentation in both human and mouse HCC cells, indicating a cross-species consistency of its antigen-presentation inhibitory effect. This finding not only complements our mechanistic understanding of impaired antigen presentation in HCC, but also suggests that endogenous tumor molecules can actively regulate the initial activation of CD8⁺ T cells, rather than merely exerting indirect effects through shaping an immunosuppressive TME. Traditionally, the primary activation of CD8⁺ T cells has been considered dependent on professional antigen-presenting cells, such as dendritic cells; however, recent studies increasingly indicate that tumor-intrinsic factors themselves can directly participate in and modulate this process, beyond the scope of microenvironmental suppression [13].

By depleting CD8⁺ T cells in vivo and establishing orthotopic liver tumor models, we found that the regulatory effect of NRGN on tumor progression is highly dependent on the presence of CD8⁺ T cells. Under conditions with intact CD8⁺ T cells, Nrgn knockdown markedly inhibited tumor growth and prolonged mouse survival, whereas the suppressive effect was largely abolished upon CD8⁺ T cell exhaustion. These results suggest that NRGN primarily promotes tumor progression by attenuating CD8⁺ T cell-mediated antitumor immunity. To specifically assess CD8⁺ T cell-mediated antitumor responses in vitro, we employed the OVA (ovalbumin) model, in which tumor cells express exogenous OVA antigen that is presented via MHC-I molecules and recognized by antigen-specific CD8⁺ T cells. This model is widely used to study tumor antigen presentation and CD8⁺ T cell activation [31, 32]. In the OVA-OT-I co-culture system, Nrgn knockdown enhanced SIINFEKL-H-2Kb expression and increased OT-I CD8⁺ T cell cytotoxicity, effector molecule secretion, and pro-apoptotic activity. Importantly, blocking antigen presentation or interfering with H-2Kb function reversed these effects. Together, these data demonstrate that NRGN suppresses CD8⁺ T cell-specific effector functions by inhibiting MHC-I-dependent antigen presentation, with antigen-specificity and well-defined functional consequences.

Mechanistic investigations further revealed that NRGN regulates antigen presentation through the IRF8-MHC-I axis. Transcriptomic analysis and in vitro validation showed that Nrgn knockdown significantly upregulated IRF8 expression, whose protein levels were positively correlated with HLA-A/B/C and β2M, suggesting that IRF8 serves as a critical hub linking NRGN to changes in antigen-presentation phenotypes. Moreover, IRF8 overexpression was able to rescue the downregulation of MHC-I and β2M induced by NRGN overexpression. Originally cloned as an IFNγ-inducible nuclear protein, IRF8 binds specific DNA motifs within classical MHC-I genes [33], and previous studies have reported that IRF8 silencing markedly reduces the expression of antigen-processing and presentation machinery components, thereby impairing dendritic cell-mediated antigen presentation [34]. Using dual-luciferase reporter assays, we confirmed that IRF8 directly binds the HLA-A and B2M promoter regions, providing a transcriptional explanation for its positive regulation of antigen presentation.

In vivo, orthotopic liver tumor models demonstrated that the antitumor effects of Nrgn knockdown were substantially attenuated in the absence of IRF8, accompanied by reduced CD8⁺ T cell infiltration and effector molecule production, further confirming IRF8’s central role in NRGN-mediated regulation of antigen presentation and immune evasion. Notably, NRGN does not regulate IRF8 transcription directly; rather, it modulates IRF8 expression post-transcriptionally by affecting mRNA stability. Actinomycin D assays revealed that NRGN decreases IRF8 mRNA stability in an FMR1-dependent manner. RNA pull-down, RIP, and multi-omics analyses collectively demonstrated for the first time that FMR1 binds the 3′UTR of IRF8 mRNA and reduces its stability, establishing the NRGN-FMR1-IRF8 axis as a key post-transcriptional regulatory mechanism. Previous reports have similarly implicated FMR1 as an mRNA stability regulator; for instance, FMR1 reduces RAC1 mRNA stability to suppress metastasis in acral and cutaneous melanoma [35], and binds the 3′UTR of PSD-95 mRNA to modulate its stability [36]. Furthermore, NRGN stabilizes FMR1 protein by inhibiting MYCBP2-mediated ubiquitination, thereby enhancing FMR1’s negative regulation of IRF8 mRNA. MYCBP2, a known HCC tumor suppressor, suppresses HCC progression by promoting HNF4α ubiquitination and degradation [37]. This mechanism not only explains how NRGN modulates IRF8 without directly binding RNA, but also provides a novel perspective on how tumor cells can indirectly reshape antigen-presenting capacity via protein homeostasis.

In vivo therapeutic experiments demonstrated that NRGN intervention markedly enhanced the response of orthotopic liver tumors to PD-1 blockade and synergistically promoted CD8⁺ T cell infiltration and effector function recovery. Coupled with the observed upregulation of β2M, these findings suggest that NRGN-mediated impairment of antigen presentation not only limits the initial activation of CD8⁺ T cells but also diminishes the efficacy of immune checkpoint blockade therapy. Accordingly, targeting NRGN or restoring the IRF8-MHC-I axis may represent a potential strategy to improve immunotherapeutic responses in HCC.

Several limitations of this study should be noted. First, the majority of mechanistic validation was performed in vitro and in murine models, which may not fully capture the heterogeneity of clinical HCC samples. Second, the precise effects of NRGN on MHC-I endocytosis, folding, and trafficking remain incompletely understood, providing avenues for further mechanistic investigations.

In summary, this study systematically demonstrates that NRGN restricts CD8⁺ T cell activation through the IRF8-MHC-I antigen presentation axis. By elucidating its role at molecular, phenotypic, and functional levels, our findings highlight NRGN as a critical mediator of immune evasion in HCC. These results provide a theoretical basis for combining NRGN-targeted strategies with immunotherapy and further expand our understanding of the mechanisms underlying impaired antigen presentation.

## Conclusion

NRGN suppresses antigen presentation via the FMR1-IRF8-MHC-I axis, thereby impairing CD8⁺ T cell-specific effector functions and promoting immune evasion in HCC. NRGN knockdown enhances MHC-I/β2M expression, restores CD8⁺ T cell activity, and increases sensitivity to PD-1 blockade. These findings suggest that NRGN may contribute to defective antigen presentation in HCC and indicate that modulation of the NRGN–IRF8–MHC-I axis could represent a potential strategy for improving immunotherapeutic responses in this context.

## Supplementary Information

Supplementary Material 1.

## Data Availability

The data that support the findings of this study are available from the corresponding author upon reasonable request.

## Abbreviations

NRGN: Neurogranin
HCC: Hepatocellular Carcinoma
TIME: Tumor Immune Microenvironment
MHC-I: Major Histocompatibility Complex Class I
CTLs: CD8⁺ Cytotoxic T Lymphocytes
β2M: β2-Microglobulin
TME: Tumor Microenvironment
SPF: Specific-Pathogen-Free
GO: Gene Ontology
KEGG: Kyoto Encyclopedia of Genes and Genomes
GSEA: Gene Set Enrichment Analysis
PBMC: Peripheral Blood Mononuclear Cell
RIP: RNA immunoprecipitation
CHX: Cycloheximide
TCGA: The Cancer Genome Atlas
