# Supplementary material for: NRGN inhibits CD8⁺ T cell effector function by downregulating MHC-I expression through the FMR1–IRF8 axis in hepatocellular carcinoma
Source: J Exp Clin Cancer Res. 2026 Jul 15;45:212. doi: 10.1186/s13046-026-03783-7 (PMC13651206; doi:10.1186/s13046-026-03783-7)
Supplement: Supplementary file 1 [file 13046_2026_3783_MOESM1_ESM.docx]

***Supplementary material***

**NRGN inhibits CD8⁺ T cell effector function by downregulating MHC-I expression through the FMR1–IRF8 axis in HCC**

Dongjie Ye^1,#^, Jiacheng Qiu^2,#^, Haoyi Yang^1,#^, **Jinqiao Wu^1^,** Xinyu Zhang^1^, Zhu Zhang^1^, Hao Wu^1^, Yuxin Yao**^1^, Xiaoqian Wang^1,3^,** Nanhong Tang^1,3,4,5*^

^1^Department of Hepatobiliary Surgery and Fujian Institute of Hepatobiliary Surgery, Fujian Medical University Union Hospital, Fuzhou, China.

^2^Department of Laboratory Medicine, Fujian Medical University Union Hospital, Fuzhou, China.

^3^Cancer Center of Fujian Medical University, Fujian Medical University Union Hospital, Fuzhou, China.

^4^Key Laboratory of Clinical Laboratory Technology for Precision Medicine (Fujian Medical University), Fujian Province University, Fuzhou, China.

^5^Key Laboratory of Gastrointestinal Cancer (Fujian Medical University), Ministry of Education, Fuzhou, China.

^#^ These authors contributed equally to this work.

^*^ Correspondence: fztnh@fjmu.edu.cn (N. Tang)

**Table S1.** Primer sequences used for qRT-PCR in human and mouse genes

|  | Forward primer（5'-3'） | | Reverse primer（5'-3'） |
| --- | --- | --- | --- |
| *NRGN* | | GCGAGTTTTCGGGGCCACAT | GCCACCCCAGCTCCGCCAG |
| *HLA-A* | | AAAAGGAGGGAGTTACACTCAGG | GCTGTGAGGGACACATCAGAG |
| *HLA-B* | | GCCGCGAGTCCGAGAGA | GCCTTGTAGATCTGTGTGTTCC |
| *HLA-C* | | GCTCAGATCACCCAGCGCAAG | TGCAGCGTCTCCTTCCCGTTC |
| *IRF8* | | GTTCATATCCAGGCAAACGG | GTTCTAATCTTCCTGGCAACA |
| *GAPDH* | | TCTGACTTCAACAGCGACACC | CTGTTGCTGTAGCCAAATTCGTT |

|  | Forward primer（5'-3'） | | Reverse primer（5'-3'） |
| --- | --- | --- | --- |
| *Nrgn* | | CCTGCTCCAAGCCAGACGACGA | TTCCGGCCACACTCTCCGCTCT |
| *H-2k* | | GGCAATGAGCAGAGTTTCCGAG | CCACTTCACAGCCAGAGATCAC |
| *H-2d* | | TGAGGAACCTGCTCGGCTACTA | GGTCTTCGTTCAGGGCGATGTA |
| *B2m* | | ACAGTTCCACCCGCCTCACATT | TAGAAAGACCAGTCCTTGCTGAAG |
| *Gapdh* | | GCGACTTCAACAGCAACTCCC | CACCCTGTTGCTGTAGCCGTA |

**Figure S1. Differential expression of NRGN in hepatocellular carcinoma and its impact on antigen presentation-related genes.** (A) TCGA-based analysis of NRGN expression across different pathological stages of hepatocellular carcinoma (HCC). (B) TCGA-based analysis of NRGN expression across different histological grades of HCC. (C) Differential expression analysis of NRGN between tumor tissues and adjacent normal tissues in the TCGA-LIHC cohort. (D) Kaplan-Meier survival analysis comparing overall survival between patients with high and low NRGN expression in the TCGA dataset. (E) Principal component analysis (PCA) of transcriptomic profiles from Hep3B control and NRGN knockdown cells. (F) Sequencing depth across all RNA-seq samples. (G) Total gene counts detected in each transcriptomic sample. (H) Statistical analysis of expression changes in key antigen presentation-related genes identified from the transcriptomic dataset. (I) Gene Ontology (GO) enrichment analysis of differentially expressed genes derived from RNA sequencing. (J) CD8⁺ T cell migration assay using conditioned medium from NRGN knockdown and control tumor cells (n = 3). **P* < 0.05; ***P* < 0.01; ****P* < 0.001; ****P* < 0.0001; ns, not statistically significant.

**Figure S2. NRGN regulates CD8⁺ T cell-mediated antitumor immunity.** (A) Flow cytometric analysis of the efficiency of CD8⁺ T cell exhaustion in vivo (n = 3). (B-C) Immunoblot analysis of β2-microglobulin (β2M) expression following NRGN modulation in Huh7 (B) and Hep3B (C) cells (n = 3). (D-E) Flow cytometric analysis of cell surface β2M expression after NRGN modulation in Huh7 (D) and Hep3B (E) cells (n = 3). (F-G) Liver weight-to-body weight ratio (F) and liver weight (G) in the orthotopic HCC model. (H-I) Flow cytometric analysis of cell surface β2m expression following Nrgn overexpression (H) or knockdown (I) in Hep-53.4 cells (n = 3). (J) Immunohistochemical staining of Ki67 in tumor tissues (n = 5). (K) Flow cytometric analysis of tumor cell surface β2m expression across different experimental groups (n = 5). **P* < 0.05; ***P* < 0.01; ****P* < 0.001; ****P* < 0.0001; ns, not statistically significant.

**Figure S3. NRGN modulates CD8⁺ T cell effector function and antigen presentation.** (A) OT-1 mouse-derived CD8⁺ T cells were co-cultured with Nrgn-manipulated Hep53.4_OVA cells for 24 h, followed by flow cytometric analysis of CD8⁺ T cell effector function markers (CD8⁺ T cells:tumor cells = 5:1) (n = 3). (B-C) Liver weight-to-body weight ratio (B) and liver weight (C) in orthotopic HCC models established in C57BL/6 mice using Hep53.4 cells with Nrgn knockdown alone or combined Nrgn knockdown and β2m knockout. (D-F) Under combined αH-2Kb treatment, liver weight-to-body weight ratio (D), liver weight (E), and body weight changes (F) were assessed. (G) Histopathological evaluation of heart, liver, spleen, lung, and kidney tissues by hematoxylin and eosin (H&E) staining to assess systemic safety. **P* < 0.05; ***P* < 0.01; ****P* < 0.001; ****P* < 0.0001; ns, not statistically significant.

**Figure S4. NRGN expression correlates with IRF8 and MHC-I/β2M levels in HCC.** (A) mRNA expression levels of IRF and STAT family-related genes in the two indicated groups. (B) Volcano plot showing differentially expressed genes identified by transcriptomic analysis in control and NRGN-knockdown Hep3B cells. (C) Differential expression of IRF8 mRNA between HCC tissues and adjacent normal tissues based on the TCGA dataset. (D-F) Correlation analyses between IRF8 protein expression and B2M, HLA-B, and HLA-C protein levels in the CPTAC dataset. (G-I) Correlation analyses between IRF3 protein expression and B2M, HLA-B, and HLA-C protein levels in the CPTAC dataset. (J-K) Immunoblot analysis of MHC-I and β2M protein expression following IRF8 overexpression and interferon stimulation in Huh7 (J) and Hep3B (K) cells (n = 3). (L) Flow cytometric analysis of cell surface MHC-I expression in Huh7 cells following NRGN overexpression with or without IRF8 restoration (n = 3). (M) Immunoblot analysis confirming IRF8 knockout efficiency in Hep3B cells (n = 3). **P* < 0.05; ***P* < 0.01; ****P* < 0.001; ****P* < 0.0001; ns, not statistically significant.

**Figure S5. NRGN regulates IRF8 expression through FMR1.** (A) Western blot analysis of protein extracts from tumor tissues of 12 patients with HCC. (B) Immunoblot analysis confirming IRF8 knockout efficiency in Hep53.4 cells (n = 3). (C-E) Liver weight (C), liver weight-to-body weight ratio (D), and overall survival (E) in orthotopic HCC models established in C57BL/6 mice using Hep53.4 tumor cells with Nrgn modulation combined with IRF8 knockout. (F-G) Flow cytometry analysis of intracellular IFN-γ and TNF-α MFI in CD8⁺ T cells in the TME across four groups (n = 5). (H) Differential expression analysis of FMR1 mRNA between HCC tissues and adjacent normal tissues based on the TCGA dataset. (I) Correlation analysis between FMR1 and IRF8 protein expression in the CPTAC dataset. (J) Immunoblot analysis confirming FMR1 knockout efficiency in Huh7 cells (n = 3). (K) Tandem mass spectrometry identification of the FMR1 protein. **P* < 0.05; ***P* < 0.01; ****P* < 0.001; ****P* < 0.0001; ns, not statistically significant.

**Figure S6. NRGN suppresses anti-tumor immunity and promotes tumor growth via FMR1. (A-B)** Actinomycin D assay evaluating the effect of NRGN intervention combined with FMR1 knockout on IRF8 mRNA stability in Huh7 (H) and Hep3B (I) cells (n = 4). **(C)** Representative images of orthotopic liver tumors in C57BL/6 mice established with Nrgn-modulated Hep53.4 cells combined with FMR1 knockout (n = 5). **(D-F)** Tumor weight (D), tumor volume (E) and Liver tumor weight-to-body weight ratio (F) in the orthotopic liver cancer model. **(G-H)** Flow cytometry analysis of intracellular IFN-γ (G) and GZMB (H) MFI in CD8⁺ T cells in the TME across four groups (n = 5). (I–J) Flow cytometric analysis of the percentages of NK cells (I) and CD4⁺ T cells (J) in the tumor immune microenvironment across four groups (n = 5). **P* < 0.05; ***P* < 0.01; ****P* < 0.001; *****P* < 0.0001; ns, not significant.

**Figure S7. NRGN shapes the immune microenvironment and tumor growth in HCC.** (A-B) Co-IP analysis of the endogenous interaction between NRGN and FMR1 in human HCC tissues (A) and control liver tissues (B). (C-D) Co-IP analysis of the endogenous interaction between FMR1 and MYCBP2 in human HCC tissues (C) and control liver tissues (D). (E) Tandem mass spectrometry identification of the MYCBP2 protein. (F-G) Immunoblot analysis confirming MYCBP2 knockdown efficiency (n = 3). (H-I) Flow cytometric analysis of TNF-α (H) and GZMB (I) MFI in CD8⁺ T cells within the tumor immune microenvironment across four experimental groups (n = 5). (J-K) Liver tumor weight (J) and body weight changes (K) in the orthotopic HCC mouse model. (L) Histopathological evaluation of heart, liver, spleen, lung, and kidney tissues by hematoxylin and eosin (H&E) staining to assess systemic safety. **P* < 0.05; ***P* < 0.01; ****P* < 0.001; ****P* < 0.0001; ns, not statistically significant.
